# Personalized pulse wave propagation modeling to improve vasopressor dosing management in patients with severe traumatic brain injury

**DOI:** 10.1371/journal.pcbi.1013501

**Published:** 2025-09-15

**Authors:** Kamil Wolos, Leszek Pstras, Urszula Bialonczyk, Malgorzata Debowska, Wojciech Dabrowski, Dorota Siwicka-Gieroba, Jan Poleszczuk

**Affiliations:** 1 Laboratory of Mathematical Modeling of Physiological Processes, Nalecz Institute of Biocybernetics and Biomedical Engineering, Polish Academy of Sciences, Warsaw, Poland; 2 Department of Anesthesiology and Intensive Therapy, Medical University of Lublin, Lublin, Poland; University of Southern California, UNITED STATES OF AMERICA

## Abstract

This study investigates whether examining the shape of arterial pulse waves and fitting to them a physiology-based mathematical model of pulse wave propagation can provide additional insights into the state of the cardiovascular system in patients with severe traumatic brain injury (sTBI), potentially enhancing vasopressor dosing strategies. We conducted a longitudinal study on 25 sTBI patients in an intensive care unit. Arterial pulse waves were recorded non-invasively from wrists and ankles using an oscillometric method and were used to inform a 0-1D model of the arterial blood ﬂow dynamics. Model-estimated, patient-specific cardiovascular parameters were then used in a statistical model to predict changes in the administered dose of vasopressor (norepinephrine) in the next 24 hours. The model fits to the recorded pulse waves were satisfactory, with the coefficients of determination ($R^{\text{2}}$) of approximately 0.9 and the differences between the measured and model-estimated mean arterial pressure of 0.1 ± 1.0 mmHg ($R^{\text{2}}$=0.99). Except for a few patients, we found no clear association between the model-estimated parameters and norepinephrine dose at the time of pulse wave recording. Nevertheless, our predictive model achieved a balanced accuracy of 0.85 when trained and tested on the entire dataset and 0.76 when using the leave-one-out cross-validation, with 8 misclassifications among the total of 77 observations. Thus, despite the known inter-patient variability of hemodynamic response to vasopressors, the proposed method allowed predicting the direction of norepinephrine dose changes in the next 24 hours with satisfactory accuracy. Subject to further studies and extensive validation, our approach could inform a decision-support tool for optimizing vasopressor dosing on a per-patient basis.

## Authors summary

Hypotension is a dangerous complication in patients with severe traumatic brain injury (sTBI), making precise control of systemic blood pressure of utmost importance. To maintain blood pressure at prescribed levels, physicians often administer vasopressors (for example, norepinephrine). In current clinical practice, the vasopressor dose is frequently adjusted by trial and error, guided by continuous monitoring of the patient’s hemodynamic state. This approach is time-consuming and likely sub-optimal, highlighting the need for more efficient methods to guide vasopressor therapy. In this study, we investigated whether non-invasive peripheral pulse wave recordings, combined with mathematical modeling of cardiovascular dynamics, could help predict changes in vasopressor dosing. Our results show that by incorporating personalized cardiovascular parameters into a statistical predictive model, it is possible to predict - with satisfactory accuracy - the direction of change (or lack thereof) in the administered norepinephrine dose within the next 24 hours. With further research and rigorous validation, this approach may support more effective vasopressor management using easily available, non-invasive clinical data.

## Introduction

Severe traumatic brain injury (sTBI) can lead to permanent disability or death, and its treatment remains a challenge [1,2]. Among the many complications associated with sTBI, hemodynamic instability, particularly hypotension, is of utmost importance [3]. An abnormally low blood pressure poses a substantial risk to patients with sTBI, exacerbating cerebral ischemia and further compromising neurological function [4,5]. To avoid hypotension in sTBI patients, clinicians commonly use vasopressors, particularly norepinephrine (NE) [6] which, through various mechanisms, mainly peripheral vasoconstriction [7], leads to an increase in arterial blood pressure. Currently, adjusting vasopressor doses in critically ill patients is an iterative process based on observing mid- to long-term changes in vital physiological parameters such as mean arterial pressure (MAP) or systolic pressure (SP). According to the guidelines for sTBI patients, to decrease mortality and improve outcomes, MAP should be maintained above 80 mmHg [6], whereas SP should be maintained above 100 mmHg in patients aged 50–69 years, and above 110 mmHg in other patients [8]. However, such an iterative, often trial-and-error process might be far from optimal, carrying the risk of transient sub-optimal dose levels, which may lead to inadequate perfusion followed by hypoperfusion-related complications or to inadvertent overdosing, resulting in other adverse effects [9]. For instance, a study on 1093 ICU patients showed that their MAP was within the target range only in 43% of time (on average), while being below and above the target in 9% and 48% of time, respectively [10]. Therefore, there is a clear need to develop new tools that would help guide the process of vasopressor dosing in sTBI patients, to ensure the best possible treatment outcomes and minimize mortality.

Several approaches to the problem of guiding vasopressor dose adjustments in critically ill patients using mathematical modeling can be found in the literature. Bighamian et al. introduced a latency-dose-response cardiovascular model to predict MAP and other cardiovascular parameters in response to epinephrine dosing [11,12], employing phenomenological equations with parameters personalized for both normotensive and hypotensive patients, as well as piglets. Yapps et al. employed a logistic regression model to predict hypotension events using data on MAP and vasopressor dosing, either taken from a public database (MIMIC II) or collected in surgical ICU patients [13]. Tang et al. demonstrated a model for MAP prediction based on current MAP, heart period, NE infusion rate, and respiratory rate [14], which was later extended and validated using data from septic patients [15]. Recently, Kao et al. proposed a lumped-parameter cardiovascular model that incorporates baroreﬂex feedback and a dynamic dose-response model of vasopressors, validated using data from piglets receiving phenylephrine [16]. Also, machine-learning approaches have been explored to predict vasopressor requirements in critically ill patients [17–20]. However, none of the above studies addressed patients with sTBI.

Interestingly, Johnston et al., who investigated the pharmacodynamics and pharmacokinetics of dopamine and norepinephrine in a group of eight patients with moderate to severe head injury, found that the pharmacodynamics of NE seem to be unpredictable in these patients, showing no significant correlations between NE dose levels or plasma concentrations and MAP, cardiac index (CI), or systemic vascular resistance index (SVRI) [21]. While higher NE doses were generally associated with higher MAP and higher SVRI, they found no correlations between changes in plasma NE and changes in MAP, CI, or SVRI, which suggests inter-patient variability of hemodynamic response to NE. Therefore, it appears difficult to quantitatively predict the exact hemodynamic effects of increasing the dose of NE.

This apparent unpredictability of cardiovascular response to NE may stem from the fact that current methods primarily rely on analyzing only a few standard hemodynamic parameters, which may not be sufficient. The arterial pulse waveform can provide more detailed information on the status of the cardiovascular system, as it is the sum of the forward and reflected pulse waves, with reflections occurring wherever there is a change in blood flow, i.e., at vessel bifurcations or where the stiffness of the arteries changes [22]. Analysis of the pulse waveform can therefore provide additional information about the state of the arterial system [22]. For example, it can help assess arterial stiffness and evaluate the effects of vasoactive drugs [23,24]. Our goal was to determine whether information obtained from personalized pulse wave propagation modeling [25–29] could be useful in predicting the direction of change (or lack thereof) in the administered NE dose in sTBI patients.

## Materials and methods

### Ethics statement

The study was approved by the Bioethical Committee at the Medical University of Lublin, Poland (KE-0254/253/2020) and was performed in accordance with the Declaration of Helsinki and all applicable regulations. Written informed consent was obtained from each patient or their legal representative in case the patient was unconscious and/or under sedation at the time of enrolment in the study.

### Study subjects

The study involved a cohort of 25 sTBI patients routinely treated at the intensive care unit; see Table 1 for group characteristics. Each patient was continuously monitored using a hemodynamic monitor, and based on its readings, especially MAP, and the patient’s overall clinical condition, clinicians manually adjusted the vasopressor doses. All dose changes, along with the times of those changes, were documented. All patients received NE. Five patients received additionally dobutamine. Due to missing data on NE dosage, we had to exclude data from 3 patients. Data from further 2 patients were excluded due to poor quality of the recorded pulse waves. Thus, the final analysis is based on data from 20 patients.

**Table 1:** Characteristics of the studied patients and administered drugs.

	Unit	All patients (n = 25)	Analyzed patients (n = 20)
Male gender		18 (72%)	16 (80%)
Age	years	50.5 ± 19.5	50.7 ± 20.1
Body mass	kg	84.9 ± 16.6	85.7 ± 16.4
Height	cm	174.1 ± 9.5	175.0 ± 9.7
Norepinephrine		20 (80%)	16 (80%)
Norepinephrine + Dobutamine		5 (20%)	4 (20%)

Data are reported as frequencies (percentages) or means ± standard deviations.

### Pulse wave recordings

**Fig 1:**
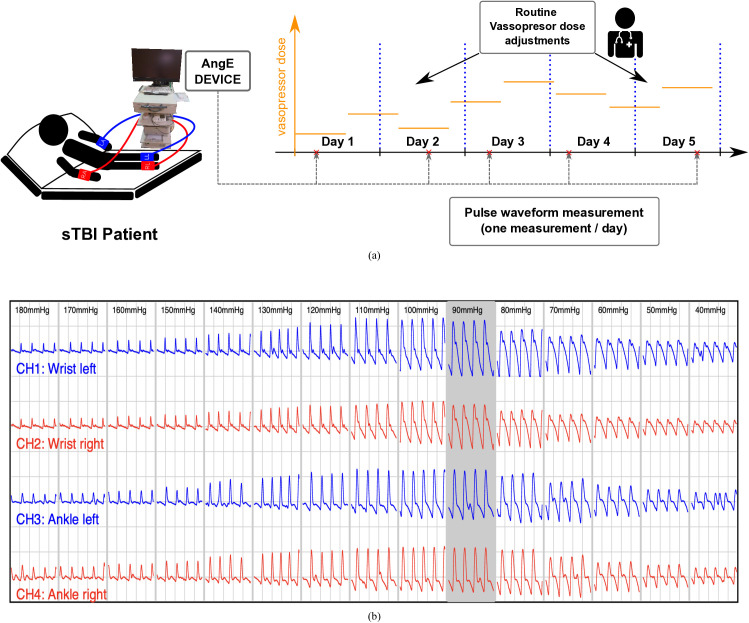
Study protocol and exemplary oscillometric waves recorded by the AngE device. (a) During the treatment (up to 5 days) one recording of pulse waves was taken each day (at various times of day) using the AngE device, while the vasopressor dose was routinely adjusted. (b) Exemplary pulse waves recorded by the AngE device.

Arterial pulse waves were recorded once per day simultaneously on both wrists and ankles (four sites in total) using the AngE device (SOT Medical, Austria), which can perform simultaneous oscillometric recordings of pulse waves at multiple sites along with electrocardiogram (ECG); see Fig 1A for an overview of the measurement protocol. The device initially inflates the cuffs to 180 mmHg and records the pressure oscillations in all cuffs for 5 seconds. The pressure in all cuffs is then reduced by 10 mmHg and the 5-second recording is repeated. This process continues until the cuff pressure reaches 40 mmHg. Thus, the total duration of a single recording is about 2 minutes; see Fig 1B for exemplary pulse wave recording. The recorded oscillations represent changes in the cuff pressure caused by the pulsatile changes of arterial volume under the cuff (attenuated to some extent), and hence for cuff pressure close to the diastolic pressure the shape of the obtained wave may be treated as relatively closely resembling the shape of the arterial volume wave [30,31]. After the recording, for each cuff, the device selects the 5-second fragment of the wave with the highest amplitude, potentially recorded at different pressure levels in different cuffs (hence at different times). For our analysis, we took from all cuffs the pulse wave fragments corresponding to the same cuff pressure level (the closest to the average level selected by the device) to analyze the waves recorded simultaneously at all sites. For each recording site, we then averaged all beats from the selected fragment of the recorded wave to obtain a single arterial volume waveform. We did not observe any significant wave profile changes following the averaging procedure. Such average waveforms from all four recording sites were used in the model optimization step (see further sections).

Note that in order to obtain the waveform that would most closely reﬂect the shape of the true arterial volume waveform, one should perform the recording with the cuff pressure kept at a level slightly below the local diastolic pressure (DP), since cuff pressures above DP lead to temporary occlusion of the arteries and hence lead to some distortion of the recorded waves [30,31]. However, at low cuff pressures, the amplitude and the overall quality of the recorded wave are much lower, which in some cases makes it difficult to identify individual beats and ultimately makes the averaged shape of the waveform less reliable (especially in patients with low pressure amplitude). Therefore, in this study, we decided to use the waves with the highest amplitude (as mentioned earlier), which were recorded at cuff pressure close to MAP, thus somewhat higher than DP. In other words, we used waveforms whose shape may differ slightly from the shape of actual arterial volume waves, but this enabled us to eﬃciently identify individual beats and hence obtain the averaged waveforms that could be used in our framework. Moreover, even if there was no occlusion of the arteries below the cuff, the waveform observed at the cuff level could still be slightly different from the actual arterial volume waveform due to the fact that the tissues between the arteries and the cuff (including the artery wall itself) may transmit the volume changes to a different extent at different pressure levels (in all cases largely attenuated).

Note also that in this study, we fit the model-simulated volume waveforms from a specific artery under the cuff (e.g., the radial artery) to the cuff-recorded waveforms, which in fact, reflect blood volume changes not in one specific artery but in all arteries below the cuff (e.g., also in the ulnar artery). This is another simplification of our approach, in which we assumed that the shapes of the volume waveforms in the radial and ulnar arteries are relatively similar to each other, and hence we treated the waveform observed at the cuff level as corresponding to the volume waveform of the radial artery. Moreover, given the relatively low changes in the diameter of the radial artery along its length, we assume that the volume waveform of the fragment of the radial artery under the cuff is equivalent to volume waveforms in other parts of the radial artery, and hence, for simplicity, we analyze model-simulated volume waveform of the whole radial artery (similarly for the anterior tibial artery for the recordings at the ankle cuffs).

### Other data obtained from the AngE device

In addition to recording the oscillometric waves, the AngE device also provides a number of cardiovascular parameters, some of which were used in our analysis. These include information on heart rate (HR) and blood pressure, i.e., systolic pressure (SP), diastolic pressure (DP), and mean arterial pressure (MAP), as well as parameters related to the recorded pulse waves, i.e., the duration of the rise in the pulse waveform from its foot to peak (‘rise time’), and the ratio of the duration of the rise in the pulse waveform and the duration of its fall from peak to the foot of the next wave (‘rise to fall’), calculated for the waveforms from each limb separately.

### Pulse wave propagation model

**Fig 2:**
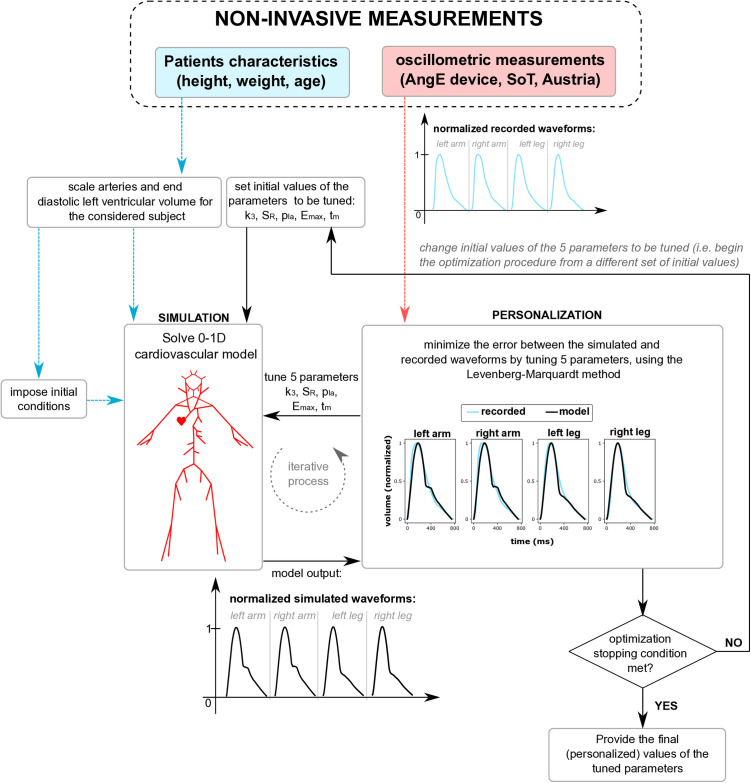
Overview of the personalization (i.e., patient-specific optimization) of the pulse wave propagation model. Fitting of the model-simulated arterial volume waveforms at four sites (wrists and ankles) to recorded waveforms by tuning selected model parameters.

We utilized a one-dimensional arterial network model composed of 71 main compliant arteries connected to zero-dimensional boundary conditions. For a detailed description of the model, please refer to our previous publications [32–34] and S1 File. The model simulates pulsatile blood flow and the corresponding blood pressure and volume waves in the whole modeled arterial tree, taking into account patient-specific characteristics, such as terminal vascular resistances, stiffness of arteries, and left-ventricular time-varying elastance function (LVTVE). We used the Levenberg-Marquardt optimization algorithm to minimize the objective (error) function by adjusting selected subject-specific model parameters. The objective function included the differences (errors) between simulated and recorded pulse waveforms (arterial volume waveforms) in four considered locations, the differences between recorded (on the cuff on the left wrist) and simulated (on the radial artery) SP and DP, and a penalty term to penalize abnormal (unphysiological) values of the simulated stroke volume (SV), according to the following equation:


$$\begin{array}{c} \text{err}=\sum_{i\in \left(\text{left}/\text{rightarm},\text{left}/\text{rightleg}\right)}\sum_{j=\text{1}}^{n}{\left\|V_{\text{norm},\text{sim};\text{i};\text{j}}-V_{\text{norm},\text{meas};\text{i};\text{j}}\right\|}^{\text{2}}\\ +\;{\left\|DP_{\text{sim}}-DP_{\text{meas}}\right\|}^{\text{2}}/\text{20}+{\left\|SP_{\text{sim}}-SP_{\text{meas}}\right\|}^{\text{2}}/\text{20}+{\left(\frac{\left(SV_{\text{sim}}-\text{70}\right)}{\text{40}}\right)}^{\text{6}} \end{array}$$ (1)

where norm, sim and meas denote normalized, simulated and measured values, respectively, ‖·‖ is a $l_{\text{2}}$-norm, n=1000 is the number of analyzed time points in the normalized simulated and measured arterial volume waveforms (V); see S1 File and Fig 2 for details (including the explanation with regard to the weights of the individual terms in our objective function).

In order to select model parameters to be optimized during the above minimization procedure, we performed a sensitivity analysis combined with an identification analysis. To this end, we first performed the Sobol’ sensitivity analysis [35,36] and calculated the first-order sensitivity indices (for each studied parameter, at multiple time points of the cardiac cycle), which quantify the direct contributions of individual parameters to the given model output variability (see Sensitivity analysis section in S1 File for details). Then we performed a parameter identification analysis using the procedure described by Olufsen et al. [37], which determines the possible pairwise correlations between the selected model parameters by calculating the relative local sensitivity matrix followed by the calculation of the inverse of the Hessian matrix (see Parameter identification section in S1 File for details). Following the above calculations, we selected five model parameters for which the analyzed outputs of the model (i.e. arterial volume waveforms at the considered locations as well as SP and DP) showed the greatest sensitivity and which were not highly correlated with each other. In particular, we selected the following model parameters: $t_{m}$ (time to the onset of constant LVTVE), $E_{max}$ (maximal value of the LVTVE function), $S_{R}$ (scaling factor for the resistances of small arteries and arterioles), $p_{la}$ (pressure in the left atrium), and $k_{\text{3}}$ (a parameter describing the stiffness of the large arteries). The values of all other parameters of the model were fixed at the levels taken from the literature as typical values for a 45-year-old man weighing 75 kg. Specifically, the values of parameters describing the stiffness of the arteries were based on a formula derived from the work of Olufsen et.al. [38], while the resistance and compliance parameters of small arteries and arterioles were taken from the work of Alastruey et al. [39]. In addition, some of the fixed parameter values were also scaled according to patient’s height. More details on the selection of individual parameters and their exact values, along with references, can be found in S1 File.

### Predictive model

To investigate the clinical utility of the model-derived, patient-specific cardiovascular parameters, we incorporated them into a statistical model to predict whether the next NE dose adjustment within 24 hours will increase the dose or not. More precisely, for each case (i.e., each time of pulse wave recording), we assigned one of two labels as follows:

- 0 (negative class) - if within 24 hours from the given pulse wave measurement, there was no dose adjustment or the first adjustment lowered the NE dose,
- 1 (positive class) - if the first dose adjustment within 24 hours from the pulse wave measurement increased the NE dose.

We used a generalized mixed linear model with a binomial link function. This model can be viewed as a logistic regression classifier suitable for data where the assumption of observation independence is violated. A detailed description of the method can be found in [40] . We considered two versions of the predictive model – a full model and a simplified model. The features considered in our full model included five patient-specific cardiovascular parameters obtained from fitting the 0-1D model to recorded pulse waves and blood pressure data $(t_{m}$, $E_{max},$ $p_{la}$, $k_{\text{3}}$, and $S_{R}$), patient characteristics (age, height, and weight), standard cardiovascular parameters measured by the AngE device (HR, SP, DP, MAP), pulse wave-related parameters provided by the AngE device (rise time of the recorded waveform from all four cuffs, and ‘rise to fall’ ratio of the recorded waveform from all four cuffs), current NE dose, and pulse wave velocity (PWV) calculated using our pulse wave propagation model (between the beginning of the ascending aorta and the end of the femoral artery, using the foot-to-foot method). To assess the utility of the parameters related to pulse waveforms (both obtained from the AngE device and estimated from our model), we also analyzed a simplified model that excluded these parameters and was based solely on patient characteristics, HR and blood pressure data, and NE dose. All features were standardized to avoid numerical problems in the process of optimizing model parameters. For both full and simplified models, the feature selection for the final model was performed using the step-wise procedure based on the Akaike Information Criterion [41]. The model performance was validated using the leave-one-out cross-validation, a suitable approach for small datasets [42]. A probability threshold of 0.5 was used, meaning that patients with a predicted probability of having an increased vasopressor dose above 0.5 were classified as belonging to the positive class.

## Results

### Model fits to recorded pulse waveforms

**Fig 3:**
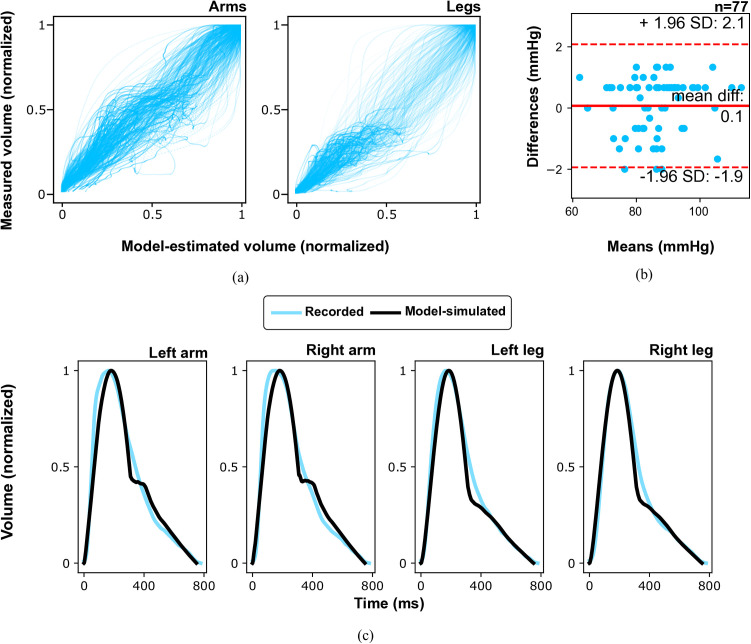
Quality of model fits to recorded data. (a) Comparison of the recorded arterial volume waveforms and corresponding in time model-simulated volume waveforms, with both the recorded and simulated waveforms normalized in amplitude (n = 77). The perfect match of the two waveforms corresponds to a diagonal line. (b) Bland-Altman plot comparing the measured and model-estimated mean arterial pressure for all analyzed cases (n = 77). (c) Typical model-simulated arterial volume waveforms (black lines) compared to the recorded (averaged) waveforms (light blue lines).

The quality of the model fits to the recorded arterial volume waveforms was satisfactory, with the mean coeﬃcients of determination ($R^{\text{2}}$) of 0.92, 0.91, 0.88, and 0.90 for the left arm, right arm, left leg, and right leg, respectively; Fig 3A shows the plots comparing all normalized model-estimated and recorded arterial volume waveforms. The model accurately estimated MAP with the mean difference between the model-derived and measured values of 0.1 ± 1.0 mmHg ($R^{\text{2}}=\text{0}.\text{99}$), with no dependence on the MAP level (Pearson’s r = 0.11, p-value = 0.35), see Fig 3B. An exemplary model fit to the recorded waveform is presented in Fig 3C; see Figs A – D in S2 File for all model fits.

### Correlations between vasopressor doses and estimated parameters

**Fig 4:**
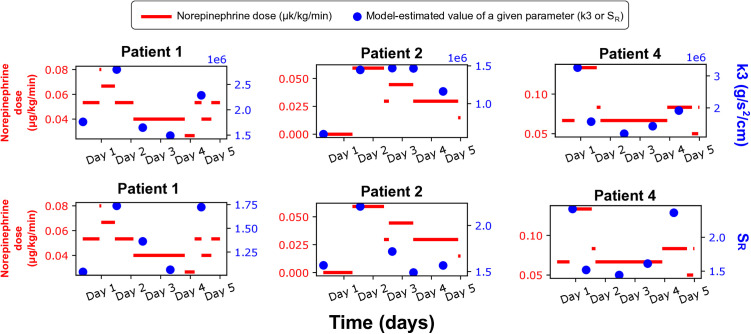
Norepinephrine dose vs patient-specific model-estimated parameters ${𝐤}_{\text{3}}$ and ${𝐒}_{R}$ for three selected patients. Shown are values of the parameters (blue dots) and norepinephrine doses (red lines).

After fitting the model to the recorded waveforms and blood pressure data, we compared the estimated patient-specific cardiovascular parameters with the NE doses at the times of pulse wave recordings. We found no correlations between the model parameters and NE doses neither for the entire cohort, nor for individual patients. Only in a few patients (ID =1, 2, 4) we observed some qualitative associations between the NE doses and parameters $k_{\text{3}}$ and $S_{R}$, see Fig 4. Similarly, we compared SP and DP (both estimated by the model and measured by AngE). In this case, we also did not observe any correlations, except for patient ID = 6, in whom SP and DP values also increased with increasing NE dose during treatment (see Figs A and B in S3 File).

### Prediction of vasopressor dose change

The dataset used in our analysis consisted of 88 observations (i.e., separate pulse wave recordings) from a group of 20 patients, among which for 77 observations we were able to assign a label 0 or 1 (the remaining observations did not have sufficient follow-up time of 24 hours). During the step-wise feature selection for our predictive model (full model), 10 features were found to be associated with the target outcome; see Table 2. Notably, these features included two model-estimated cardiovascular parameters: $p_{la}$, and $E_{max}$, that is left-atrial pressure, and maximal value of the LVTVE function, respectively. When trained and tested on the entire dataset, the model achieved a balanced accuracy of 0.85 (accuracy 0.90). When validated using the leave-one-out cross-validation (LOOCV), the model’s balanced accuracy was 0.76 (accuracy 0.82). The confusion matrices for the full model trained and tested on the entire dataset or tested using LOOCV are presented in Table 3.

**Table 2:** The coefficients of the full and simplified models predicting norepinephrine dose changes. The features included in the presented models were based on the step-wise feature selection procedure using the Akaike Information Criterion.

Feature	Full model		Simplified model		Description
	Coefficient	P-value	Coefficient	P-value	
Intercept	-1.82	<0.001	-2.48	0.05	
Norepinephrine dose	-1.80	<0.05	-2.77	<0.01	
HR	4.24	<0.05	0.06	<0.01	Heart rate
Height	1.21	<0.05	0.06	0.10	
Weight	-0.68	0.12	-0.05	0.07	
MAP	–	–	-0.11	<0.01	Mean arterial pressure
$E_{max}$	-1.48	0.09	–	–	Maximal value of the LVTVE function
$p_{la}$	-1.69	<0.05	–	–	Left-atrial pressure
Rise time (right wrist)	3.41	<0.01	–	–	Duration of the rise in the pulse waveform from the right arm (from foot to peak)
Rise to fall (right wrist)	-1.92	0.14	–	–	The ratio of the duration of the rise in the pulse waveform in the right arm (from foot to peak) and the duration of its fall (from peak to the foot of the next wave)
DP	-2.11	<0.01	–	–	Diastolic pressure measured with the left arm cuff
PWV	-1.24	<0.05	–	–	Pulse wave velocity calculated from the model

**Table 3:** Confusion matrices for the predictions of the full model trained and tested on the entire dataset or validated using the leave-one-out cross validation (LOOCV).

	Entire dataset		LOOCV	
	Predicted Negative	Predicted Positive	Predicted Negative	Predicted Positive
Actual Negative	53	2	49	6
Actual Positive	6	16	8	14

Table 2 also shows the features selected for the simplified model, which excluded pulse wave-related parameters (either derived from the AngE device or obtained from the pulse wave propagation model). This simplified model exhibits lower balanced accuracy compared to the full model, both when trained and tested on the entire dataset (0.75) and when validated using LOOCV (0.67). A detailed comparison of the performance metrics of the two models is provided in Table 4.

**Table 4:** Metrics of performance of the full predictive model trained and tested on the entire dataset (see the confusion matrix in Table 3), along with the comparison with the simplified model (also trained and tested on the entire dataset).

	Full model	Simplified model	Advantage of the full model (%)
Accuracy	0.90	0.83	7.8
Sensitivity/True Positive Rate/Recall	0.73	0.55	33.3
Specificity/True Negative Rate	0.96	0.95	1.9
Balanced Accuracy	0.85	0.75	13.4
False Positive Rate	0.04	0.05	-33.3
Precision	0.89	0.80	11.1
F1-score	0.80	0.65	23.3

In S4 File, we illustrated the predictions of the NE dose adjustments corresponding to all pulse wave recordings in all analyzed patients (the results from the full model trained and tested on the entire dataset). In some cases, despite the incorrect prediction, the model correctly anticipated the overall trend in dose adjustments (see patients with ID 7 and 18). Interestingly, in the case of the patient with ID = 18, the prediction in Day 1 was a false negative (see Fig A in S4 File) with a very low probability value (0.02). This prediction was false because at the next dose adjustment the NE dose was increased; however, this dose increase was only transient, and soon the dose was decreased to much lower levels.

For some observations from the last day, we did not have information about the potential changes in NE dose in the following 24 hours. These cases were excluded from our main analysis, but we proceeded with predictions for these cases anyway, even if their accuracy could not be assessed. In each of these cases, the model predicted that the dose would remain constant or be decreased within the next 24 hours, which seems to be expected, as one would expect the patient’s condition to improve after 4–5 days of treatment.

## Discussion

In our study on patients with severe traumatic brain injury, we explored the potential of using information extracted from arterial pulse waveforms (either directly or through pulse wave propagation modeling) to predict changes in NE dose administration that will likely be needed within the next 24 hours. We showed that knowledge of selected patient-specific cardiovascular parameters derived from the pulse waveforms, which are not measured during standard treatment procedures, appears to be useful for predicting changes in the administered NE dose in these patients possibly providing some insights into the mid- to long-term effects of the current NE dose considering the current state of patient’s cardiovascular system, thus providing the basis for a tool that could be potentially used to guide vasopressor dosing, in particular with regard to adjusting the doses sooner.

### Key results

The proposed statistical model predicting NE dose adjustments (full model) is based on 10 features, including three features derived from the 0-1D cardiovascular model ($E_{max}$, $p_{la}$, and PWV) and two features computed directly from the recorded pulse waveform (‘rise time’ and ‘rise to fall’ ratio in the right arm). Integrating the parameters available from the AngE measurements with parameters obtained from the cardiovascular model optimization enhances the performance of our predictive model. Specifically, the model that included pulse-wave-derived features (full model), exhibited a 33.3% higher sensitivity (i.e., the rate of correct predictions of NE dose increases) and 13.4% higher balanced accuracy compared to the model without those features (simplified model) when trained and tested on the entire dataset; see Table 4 for more details. The markedly higher sensitivity while maintaining similar specificity is essential from our perspective, as accurately predicting that the vasopressor dose is likely to be increased within the next 24 hours seems to be particularly important when focused on preventing episodes of hypotension in sTBI patients.

All analyzed patients were treated with NE; four patients were also given dobutamine. The effects of NE include, among others, vasoconstriction, which leads to increased arterial stiffness [43]. In line with this, the proposed predictive model accounts for the model-estimated PWV, a standard marker of arterial stiffness [44]. NE also improves end-systolic elastance [45] and increases the heart rate [46] and systemic diastolic blood pressure [47], all of which are features of our full model (end-systolic elastance is described by $E_{max}$ ). According to our predictive model, higher values of $p_{la}$ and PWV are associated with a decreased likelihood of requiring an increased NE dose within the next 24 h. This is not unexpected, given that these parameters are related to blood pressure. The fact that they were selected as predictors for our predictive model suggests that they may carry information on the current state of the cardiovascular system that may not be captured by standard cardiovascular parameters. Interestingly, our full predictive model does not include MAP as a predictor, although it is the main parameter used by clinicians to determine vasopressor dosage. However, MAP can be estimated from systolic blood pressure (SP) and diastolic pressure (DP) using a linear relationship, such as MAP = 2/3DP + 1/3SP [48], which may explain the absence of MAP and presence of DP in our full model. Indeed, during our various tests, when we removed SP and DP from the set of potential predictors, MAP emerged as an important predictor.

### Secondary findings

We also compared the patient-specific cardiovascular parameters estimated during model optimization (i.e., $k_{\text{3}}$, $S_{R}$, $t_{m}$, $E_{max}$ and $p_{la}$) with the NE doses. In general, we did not find any relationship between NE doses and the estimated parameter values. As shown in previous studies [21,49] the pharmacodynamics of NE appear unpredictable and may be due to patient variability. Such variability may explain the observed lack of relationship between the values of the estimated parameters and the administered NE doses in our cohort. However, in three patients (ID =1, 2, 4) we observed relatively similar patterns between NE doses and the values of two estimated parameters: $k_{\text{3}}$ and $S_{R}$. We believe that this was not coincidental and may be due to the fact that NE is responsible, among others, for the increase in peripheral resistance ($S_{R}$ is the scaling factor for the resistances of small arteries and arterioles) and arterial stiffness ($k_{\text{3}}$ describes the stiffness of large arteries). The fact that such relationships were not observed in other patients may be due to the effects of other vasoactive substances, e.g., endogenous catecholamines, which can disrupt the relationship between NE dose and the discussed parameters [50], or due to impact of other drugs not considered in our study, or due to neural mechanisms controlling vascular tone (also not considered in our study).

### Other studies

The problem of optimizing the dosing of vasopressors in critically ill patients has been the subject of multiple studies. For predicting a patient’s condition or the required vasopressor dose, researchers mainly use standard hemodynamic parameters that are routinely measured during standard treatment procedures and validate their methods using data from ICU patients [17–20]. In the case of mathematical models of the hemodynamic response to vasopressors, the models are mainly validated using data from either healthy individuals [11,12], septic patients [14,15], or animals [16]. To our knowledge, however, no such predictive or modelling studies have focused on sTBI patients, and hence our study seems to be the first to use personalized cardiovascular parameters estimated from a pulse wave propagation model (based on non-invasive oscillometric measurements of arterial pulse waveforms) to be fully conducted on data from a cohort of sTBI patients treated with NE.

Recently, a novel approach to vasopressor dosing has been proposed by Rinehart et. al. who developed a closed-loop controller for automatized vasopressor administration (targeting MAP) in perioperative or intensive care settings [51,52].This controller has been subsequently tested by Joostens et. al. in a randomized controlled study on titrating NE in 18 sTBI patients [53], showing that in patients in whom NE was titrated in an automatically controlled manner, MAP remained within the target range 96% of time on average (over the four-hour study period), as compared to only 43% of time in the group with standard, manual NE titration. The approach by Rinehart and Joostens, i.e., automatized NE titration, is entirely different to that considered in our study, and while it offers a great perspective for a fully automatized process of vasopressor dosing (not only in sTBI patients but in other critically ill patients or in patients undergoing surgeries), it requires special or modified equipment for drug titration. On the other hand, our approach could potentially lead to improved vasopressor dosing strategies within standard care using the existing equipment. Moreover, it is possible that integrating pulse wave features in a controller targeting MAP could potentially lead to even better performance of such a controller.

### Limitations

Our study has certain limitations. First, our findings are based on relatively limited data from a small cohort of patients, with only one pulse wave recording per day at various, arbitrary times, unrelated to vasopressor dose adjustments (although despite this, our full model was able to predict with relatively high balanced accuracy (0.85) whether the NE dose would be increased within the next 24 hours). Second, the data to which the model is fitted is subject to error measurement and natural variability (especially blood pressure), and therefore it may not necessarily reflect accurately the current (average) state of the cardiovascular system. Moreover, although the model parameters that were fixed rather than adjusted appear to have little effect on the arterial volume waveforms and blood pressure, possible changes in many of these parameters could collectively have a non-negligible effect on the behavior of the modeled system and thus on the values of the estimated (adjusted) parameters, which in turn could affect the predictions in our statistical model. Furthermore, by adjusting the value of parameter $S_{R}$, we assume that the resistance of all small arteries and arterioles (the terminal 0D elements in our model) changes to the same extent (in percentage terms, from their baseline values), which is a simplification, given that vessels in different tissues and organs may respond differently to vasoactive substances (not only in terms of the magnitude of the change in their tone but even in terms of the direction of this change) and may be also subject to varying degrees of vascular autoregulation (e.g., in cerebral vessels, although in patients with sTBI cerebrovascular autoregulation is frequently impaired [54]). Also, for simplicity, in all our simulations we assumed intact cerebral vasculature, thus ignoring the fact that patients with sTBI will likely experience alterations in cerebrovascular function, which could affect the total cerebral resistance and compliance and thus could influence central and peripheral hemodynamics, potentially affecting the results of our study. Finally, the presented approach is based on recording pulse waveforms at multiple locations (to account for the possible differences in peripheral pulse waveforms between different limbs, we chose to fit our cardiovascular model to pulse waveforms from four sites, recorded oscillometrically). We want to highlight, however, that the proposed methodology could be potentially used with pulse waveform recorded only at a single site, thus not necessarily requiring recordings on four limbs. Moreover, one could use arterial volume waveforms recorded using other methods, or use arterial pressure waveforms (e.g., recorded using applanation tonometry).

## Conclusions and future work

This study provides a preliminary demonstration of the potential of pulse wave propagation modeling for sTBI patient management. Using the data collected in sTBI patients, we developed a statistical model to predict vasopressor dose increases within 24 hours with relatively high balanced accuracy (0.85). Including in this predictive model the patient-specific parameters related to arterial stiffness and heart function, estimated from the pulse wave propagation model, turned out to provide a higher model sensitivity compared to the model without pulse wave-related features. The proposed framework could be potentially used to adjust vasopressor doses not only based on the current condition of the patient (as is currently done) but also (to some extent) taking into account predictions of vasopressor dose adjustments that are likely to be needed in the future. However, it is not obvious yet how exactly the vasopressor doses could be adjusted taking into account the predictions of the proposed model and whether this would lead to improved patient outcomes. Moreover, here we proposed a model for predicting whether the vasopressor dose will be increased by clinicians within 24 hours from the given time (the time of recording the pulse wave and blood pressure), which can be treated only as indirect information of the likely future state of the patient. Ideally, a similar framework should be developed with a model predicting directly the future state of the patient (e.g., MAP). Future studies should investigate these aspects using more comprehensive models (with various cardiovascular regulatory mechanisms) and more data, including more frequent pulse wave recordings, continuous vital parameter monitoring, and data on endogenous catecholamines.

## Supporting information

S1 File Detailed description of the mathematical model and sensitivity analysis. (PDF)

S2 File Fits of the model-simulated arterial volume waveforms to the recorded waveforms. (PDF)

S3 File Visualization of changes of systolic and diastolic pressure during the treatment. (PDF)

S4 File Visualization of the vasopressor doses and their adjustments during the observation period, along with the predictions of the statistical model (full model) with regard to the changes in vasopressor dose within the next 24 hours from the time of pulse wave recording. (PDF)
