# Supplementary material for: Personalized pulse wave propagation modeling to improve vasopressor dosing management in patients with severe traumatic brain injury
Source: PLoS Comput Biol. 2025 Sep 15;21(9):e1013501. doi: 10.1371/journal.pcbi.1013501 (PMC12527194; doi:10.1371/journal.pcbi.1013501)
Supplement: S1 File — (PDF) [file pcbi.1013501.s001.pdf]

S1 FILE: SUPPLEMENTARY MATERIAL
FOR THE ARTICLE
PERSONALIZED PULSE WAVE PROPAGATION MODELING TO IMPROVE VASOPRESSOR DOSING MANAGEMENT IN PATIENTS WITH SEVERE TRAUMATIC BRAIN INJURY

Kamil Wolos[1], Leszek Pstras[1], Urszula Bialonczyk[1], Malgorzata Debowska[1], Wojciech Dabrowski[2], Dorota Siwicka-Gieroba[2], Jan Poleszczuk[1]

[1]Laboratory of Mathematical Modeling of Physiological Processes
Nalecz Institute of Biocybernetics and Biomedical Engineering
Polish Academy of Sciences, Warsaw, Poland

[2]Department of Anesthesiology and Intensive Therapy,
Medical University of Lublin, Lublin, Poland

In this supplement, we present in more detail the methods used in our study. First, we present a detailed description of the 0-1D cardiovascular model. Next, we describe how we selected patient-specific parameters to be used in the model optimization procedure. Finally, we describe the optimization procedure.

1 The Cardiovascular Model

The mathematical foundations of the model that we present in this section come from the work of Sterigiopulos et. al [1], Olufsen et. al. [2], and Ottesen et. al, [3], [4].

1.1 Geometry of the arterial tree

The one-dimensional arterial bifurcation tree represents 71 major human arteries, including cerebral circulation; see Fig A for more details. Each segment (artery) is modeled as a compliant axi-symmetric, tapering cylinder with impermeable walls. The geometric parameters of each segment (length L, inlet internal radius r_{in}, and outlet internal radius r_{out}) are adapted from the work of Stergiopulos and Alastruey, [1], [5], see Table A for more details. Tapering of a given vessel is described by the following equation:

$$r_0(x) = r_{\text{in}} \left(\frac{r_{\text{out}}}{r_{\text{in}}} \right)^{x/L}, \tag{1}$$

where $r_0(x)$ denotes the internal radius of a given artery at point x at the nominal pressure P_0, [2].

1.2 Blood flow through the artery

The presented mathematical model is based on the Navier-Stokes equations and describes the changes in flow $Q(t, x)$, internal cross-sectional area $A(t, x)$, and pressure $P(t, x)$ along the vessel. We assume that blood is an incompressible fluid with constant density ρ and viscosity μ, and that the flow in the artery has a Poiseuille (parabolic) velocity profile. The continuity and momentum equations, derived using standard methods (detailed in [2], [3]), are given by,

$$\frac{\partial Q(t,x)}{\partial x} + \frac{\partial A(t,x)}{\partial t} = 0, \tag{2}$$

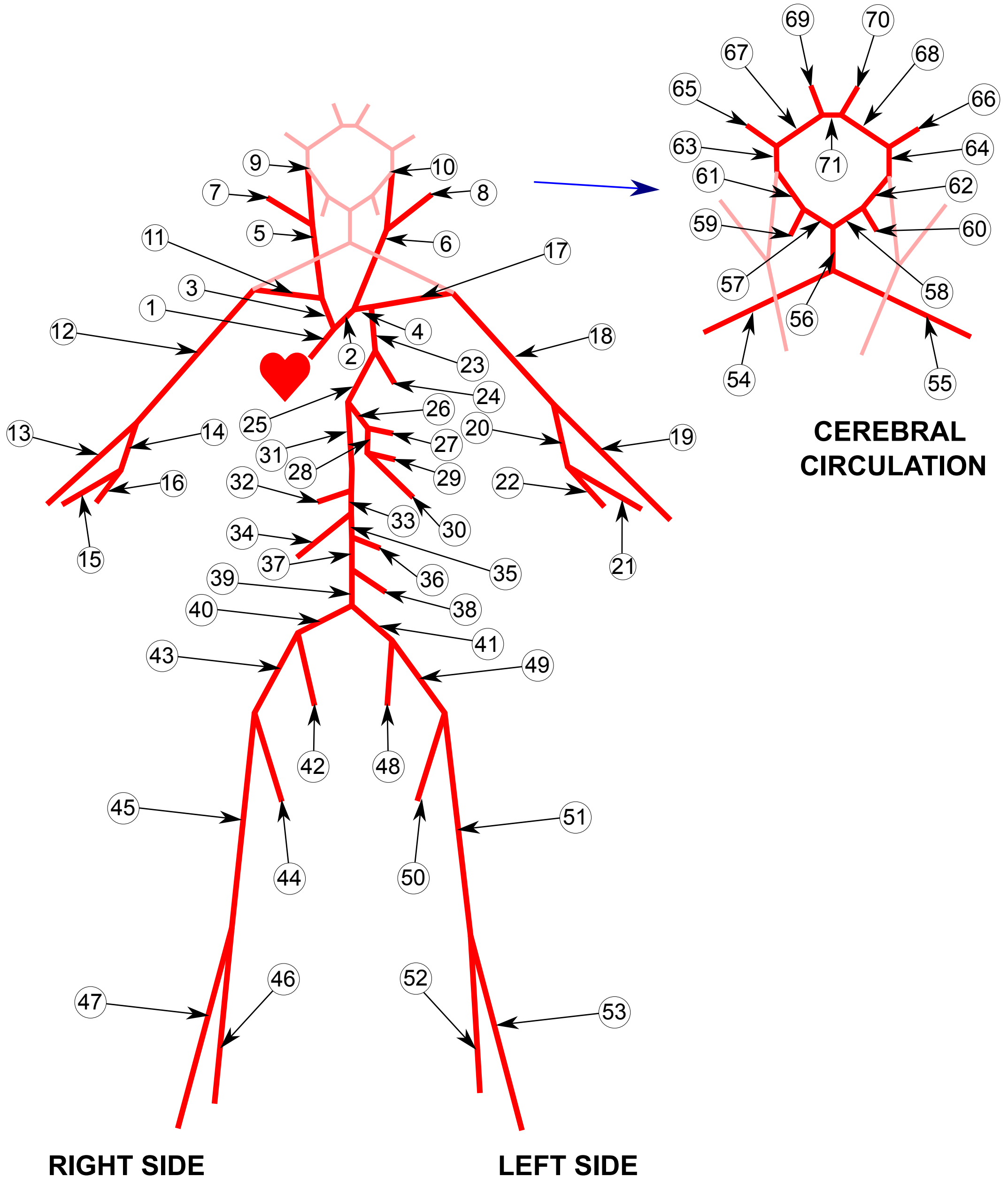

Fig A. **Overview of the modelled arterial tree.** For more information on individual arteries, see Table A.

$$\frac{\partial Q(t,x)}{\partial t} + \frac{\partial}{\partial x}\left(\frac{Q(t,x)^2}{A(t,x)}\right) + \frac{A(t,x)}{\rho}\frac{\partial P(t,x)}{\partial x} = \frac{-8\pi\mu}{\rho}\frac{Q(t,x)}{A(t,x)}. \tag{3}$$

Since in equation (3) the pressure appears only in the gradient, we need to add one more equation using P to ensure that the solution is unique. Assuming that the arterial walls are purely elastic, we can introduce the following equation, relating pressure to the internal cross-section of the artery [2]:

$$P(t,x) - P_0 = f(x)\left(1 - \sqrt{\frac{A_0(x)}{A(t,x)}}\right), \tag{4}$$

Table A: **Geometry of the modelled arterial tree and the corresponding peripheral resistances and compliances.** Length, r_{in} and r_{out} are provided in centimeters. Total peripheral resistance, $\left(R_T,\, 10^4\,\text{g/cm}^4/\text{s}\right)$ and compliance $\left(C_T,\, 10^6\,\text{cm}^4\text{s}^2/\text{g}\right)$ are provided only for terminal arteries. L and R stands for left and right, respectively. Data adapted from [1], [5].

ID	Artery name	Length	$\mathbf{r}_{\text{in}}$	$\mathbf{r}_{\text{out}}$	$\mathbf{R_T}$	$\mathbf{C_T}$
1	Ascending aorta	4	1.2	1.18	-	-
2	Aortic arch (I)	2	1.12	1.11	-	-
3	Brachiocephalic	3.4	0.62	0.61	-	-
4	Aortic arch (II)	3.9	1.07	1.06	-	-
5/6	Common Carotid R/L	17.7/20.8	0.25	0.25	-	-
7/8	External Carotid R/L	17.7	0.15	0.14	5.43	12.70
9/10	Internal Carotid (I) R/L	17.7	0.2	0.2	-	-
11/17	Subclavian (I) R/L	3.4	0.42	0.42	-	-
12/18	Subclavian (II) R/L	42.2	0.4	0.24	-	-
13/19	Radial R/L	23.5	0.17	0.14	5.28	3.52
14/20	Ulnar (I) R/L	6.7	0.22	0.22	-	-
15/21	Interosseous R/L	7.9	0.1	0.1	8.40	0.22
16/22	Ulnar (II) R/L	17.1	0.2	0.18	5.28	3.52
23	Thoracic aorta (I)	5.2	1	1	-	-
24	Intercostals	8	0.2	0.15	1.39	13.38
25	Thoarcic aorta (II)	10.4	0.68	0.65	-	-
26	Celiac (I)	1	0.39	0.39	-	-
27	Hepatic	6.6	0.22	0.22	3.64	5.13
28	Celiac (II)	1.0	0.2	0.2	-	-
29	Gastric	7.1	0.18	0.17	5.43	3.44
30	Splenic	6.3	0.18	0.17	2.32	8.01
31	Abdominal aorta (I)	5.3	0.61	0.6	-	-
32	Superior mesenteric	5.9	0.44	0.42	0.93	20.0
33	Abdominal aorta (II)	1	0.6	0.59	-	-
34/36	Renal R/L	3	0.26	0.25	1.13	16.46
35	Abdominal aorta (III)	3	0.59	0.58	-	-
37	Abdominal aorta (IV)	10.6	0.58	0.55	-	-
38	Inferior mesenteric	5.0	0.17	0.16	6.89	2.70
39	Abdominal aorta (V)	1.0	0.54	0.52	-	-
40/41	Common iliac R/L	5.8	0.37	0.35	-	-
42/48	Internal iliac R/L	5	0.2	0.19	7.96	2.34
43/49	External iliac R/L	14.5	0.32	0.27	-	-
44/50	Deep femoral R/L	12.6	0.26	0.19	4.79	3.90
45/51	Femoral R/L	44.5	0.26	0.19	-	-
46/52	Posterior tibial R/L	32.1	0.16	0.14	4.79	3.90
47/53	Anterior tibial R/L	34.3	0.13	0.12	5.60	3.33
47/53	Anterior tibial R/L	34.3	0.13	0.12	5.60	3.33
54/55	Vertebral R/L	14.8	0.14	0.14	-	-
56	Basilar artery	3	0.16	0.11	-	-
57/58	Posterior cerebral (I) R/L	0.5	0.11	0.11	-	-
59/60	Posterior cerebral (II) R/L	8.5	0.11	0.11	11.08	6.20
61/62	Posterior communicating R/L	1.5	0.07	0.07	-	-
63/64	Internal carotid (II) R/L	0.5	0.2	0.19	-	-
65/66	Middle cerebral R/L	12	0.14	0.12	5.97	11.60
67/68	Anterior cerebral (I) R/L	1.2	0.12	0.12	-	-
69/70	Anterior cerebral (II) R/L	10	0.12	0.12	8.48	8.20
71	Anterior communicating	0.3	0.07	0.07	-	-

where $A_0(x)$ is the artery's internal cross-sectional area at nominal pressure, i.e., $A_0(x) = \pi r_0^2(x)$, and the function $f(x)$ describes the elasticity of the artery wall as follows:

$$f(x) = \frac{4}{3}\left(k_1 \exp\left(k_2 r_0(x)\right) + k_3\right), \tag{5}$$

where parameters k_1, k_2 and k_3 are global subject-specific constants. Parameter k_3 describes the stiffness of large arteries, parameter k_1 describes the increasing stiffness of smaller arteries, and k_2 reflects the transition between the large, elastic arteries and smaller, less-elastic arteries [6].

1.3 Bifurcations

We assume pressure continuity and mass conservation (i.e. no blood leakage) at the vessel nodes. For a discussion on the validity of these assumptions, see [3]. If by p we denote the parent vessel, and d_1, d_2 are the daughter vessels, then the above conditions may be expressed as follows:

$$P_{\text{out},p} = P_{\text{in},d_1} = P_{\text{in},d_2}, \quad \text{and} \quad Q_{\text{out},p} = Q_{\text{in},d_1} + Q_{\text{in},d_2}. \tag{6}$$

1.4 Inflow Boundary Condition

The inflow boundary condition describes the blood ejection from the left heart ventricle and is based on the work of Suga et al. [7], [8], and Danielsen and Ottensen [4]. Changes of the pressure in the left ventricle, P_{lv}, can be described using a time-varying left-ventricle elastance function $E_{lv}(t)$:

$$P_{lv} = E_{lv}(t)\left(V_{lv}(t) - V_0\right), \tag{7}$$

where V_{lv} is the ventricular volume and V_0 is the volume of the left ventricle at zero transmural pressure. According to [4], the function $E_{lv}(t)$ may be expressed as follows:

$$E_{lv}(t) = E_{\text{min}}\left(1 - \phi(t)\right) + E_{\text{max}}\phi(t), \tag{8}$$

where the parameters E_{min} and E_{max} are minimal and maximal values of the elastance function $E_{lv}(t)$. Function ϕ is defined by the following equation:

$$\phi(t) = \begin{cases} a\sin\left(\frac{\pi t}{t_m}\right) + b\sin\left(\frac{2\pi t}{t_m}\right) & \text{for} \quad 0 \le t < t_m \\ 0 & \text{for} \quad t_m \le t < T \end{cases}, \tag{9}$$

where T is the heart period, t_m denotes the onset of constant (minimal) elastance, and parameters a and b are responsible for the shape of the $\phi(t)$. Additionally, a and b must be chosen so that $\max_{t\in[0,T]} \phi(t) = 1$. The example of the simulated left ventricle elastance is shown in Fig B.

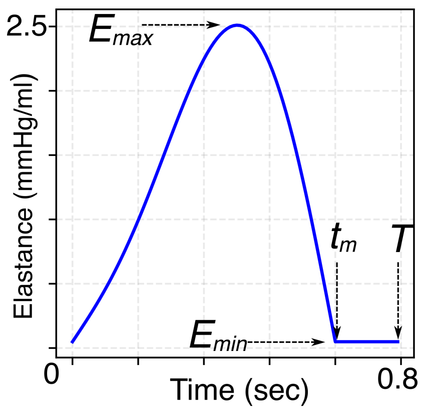

Fig B. **Left-ventricle time-varying elastance function.** $E_{\text{min}}, E_{\text{max}}$ - minimal and maximal value of the elastance function, t_m - onset of the constant (minimal) elastance, T - heart period.

The work of the left ventricle can be divided into four stages. We will begin with isovolumic relaxation. During this phase, the pressure in the left ventricle decreases. When P_{lv} is smaller than the pressure in the left atrium, P_{la}, then the mitral valve opens.

In the next phase (ventricular filling) blood flows from the left atrium to the left ventricle. This flow, Q_{la}, is described by the following equation:

$$\frac{dQ_{la}}{dt} = \frac{1}{L_{la}}\left(P_{la} - P_{lv}\right) - \frac{R_{la}}{L_{la}}Q_{la}. \tag{10}$$

Parameter L_{la} is an inertia term, and R_{la} describes the resistance against the flow from the atrium to the ventricle, caused mainly by the viscous properties of the blood. Simultaneously, due to the inflow of blood into the left ventricle, V_{lv} increases, given by the following equation:

$$\frac{dV_{lv}}{dt} = Q_{la}. \tag{11}$$

When V_{lv} is greater than end-diastolic volume V_{ed}, the mitral valve closes, and isovolumic contraction begins.

During this phase, there is no flow between the left atrium and ventricle ($Q_{la} = 0$), and P_{lv} increases. When P_{lv} is greater than the pressure in the ascending aorta, P_a, the aortic valve opens, and the last phase of the cycle (ventricular ejection) begins.

The flow between the ventricle and aorta is expressed by an equation similar to equation (10), namely:

$$\frac{dQ_{lv}}{dt} = \frac{1}{L_{lv}} (P_{la} - P_a) - \frac{R_{lv}}{L_{lv}} Q_{lv}. \tag{12}$$

The pressure P_a is taken directly from the 1-D model of the arterial tree. The volume V_{lv} decreases according to the following equation:

$$\frac{dV_{lv}}{dt} = -Q_{lv}. \tag{13}$$

At the end of this phase, some amount of blood, V_b, returns from the ascending aorta to the left ventricle, which is associated with the negative value of Q_{lv} (backflow). V_b is given by the following equation:

$$V_b = \int_{t^*}^{t} |Q_{lv}|, \quad \text{for} \quad t > t^*, \tag{14}$$

where t^* denotes the moment, when Q_{lv} becomes negative. At the time t, when $V_b > \overline{V_b}$ we end the last phase by setting $Q_{lv} = 0$, and then the cycle repeats.

1.5 Outflow boundary conditions

The outflow boundary conditions are modeled using a three-element Windkessel model [9], [10], which describes compliance and resistance effects of the vessels beyond the modeled terminal arteries:

$$R_1 R_2 C_T \frac{dQ_{\text{end}}(t)}{dt} = R_2 C_T \frac{dP_{\text{end}}(t)}{dt} + (P_{\text{end}}(t) - P_T) - (R_1 + R_2) Q_{\text{end}}(t). \tag{15}$$

In the above equation, R_1 and R_2 are proximal and distal resistances, respectively, C_T is the total compliance of the terminal vascular branch, and P_T is the reference terminal pressure. Moreover, we assume that $R_1/R_T = 0.2$, where R_T is the total terminal resistance, and $R_T = R_1 + R_2$ as in [1], [10]. Table A lists all values of C_T and R_T, adapted from [1] and [5].

1.6 Solving the model equations

The procedure of solving the model equationsis similar to the one presented in our previous work [11]. The governing equations for blood flow in the 1D domain were solved using the Lax-Wendroff scheme [12]. The inflow and outflow boundary conditions were integrated into the 1D model using the "ghost point" method [13], [14]. The inflow boundary condition was solved with the Runge-Kutta scheme, and the 3-element Windkessel models were solved using the explicit Euler method. The time step was set to $\Delta t = 2 \cdot 10^{-4}$ s, and spacial discretization was set to $\Delta x = 0.5$ cm (Courant number in radial artery for a default parameters is $C = 0.016$).

2 Model parameters

2.1 Arterial tree geometry

The procedure of personalizing the arterial tree geometry was similar to that employed in our previous publications [11], [15], [16]. The default arterial tree (Table A) represents the typical arterial geometry for a 175 cm tall man. To personalize the arterial tree for a given patient, we multiplied all nominal artery lengths as well as internal proximal and distal artery radii by a scaling factor S=H/175, where H is patient's height in cm.

2.2 Blood flow and vascular parameters

We set blood density as $\mu = 1.04 \frac{\text{g}}{\text{cm}^3}$ and kinematic viscosity as $\rho = 0.04 \frac{\text{cm}^2}{s}$ [10]. The nominal mean arterial pressure, P_0, was set to 97 mmHg [1]. With regard to parameters describing the stiffness of the arteries (equation (5)), as in [6], we set parameter k_2 to $-13.5 \frac{1}{\text{cm}}$. The other two parameters (k_1 and k_3) were considered in the sensitivity analysis discussed later (see Table B for their assumed baseline values).

2.3 Inflow boundary condition

The blood pressure in the left ventricle, P_{lv}, is computed using a time-varying elastance function $E_{lv}(t)$, see eq. (7). Since the shape of the left-ventricular elastance function (normalized for amplitude and time to peak) remains relatively constant, even in various cardiovascular diseases [17], we decided to fix the parameters describing the shape of the elastance function, $a = 0.9$ and $b = 0.25$, as in [4]. We assumed that the volume of blood backflow to the left ventricle at the end of each cycle ($\overline{V_b}$), equals 2 ml [4]. Based on the work of Parikh et. al. [18], we determined the relation between the left end-diastolic volume index (LEDVi) and the age, corrected for the body surface area (BSA), calculated using the Du Bois formula, as follows [19]:

$$BSA = 0.007184 \cdot \text{Weight}^{0.425} \cdot \text{Height}^{0.725}. \tag{16}$$

After performing linear fitting to data from [18], we obtained the following relation:

$$V_{ed} = (-0.29 \cdot \text{Age} + 81) \cdot BSA. \tag{17}$$

The parameters that describe the flow from the left atrium to the left ventricle (L_{la}, R_{la}) and from the left ventricle to the ascending aorta ($L_{lv}, R_{lv}, E_{\text{min}}, E_{\text{max}}$ and P_{la}) were considered in the sensitivity analysis described later (see Table B for their assumed baseline values).

2.4 Outflow boundary condition

Each terminal artery was connected to a three-element Windkessel model, describing the behavior of small arteries, arterioles, and capillaries downstream of the given terminal artery, characterized by the total resistance, R_T and compliance, C_T. The nominal values of these parameters for each terminal artery are presented in Table A, based on data from the literature [1], [5]. Similarly to other studies [11], [14], [20] these parameters are scaled globally, using separate scaling factors for resistances and compliances (S_R and S_C, respectively). The reference terminal pressure, P_T, was set at 15 mmHg as in our previous study [16].

3 Sensitivity analysis

The sensitivity analysis was performed to identify model parameters with the greatest impact on the shape of the pulse waveform. The following parameters were analyzed: k_1, k_3 – parameters describing the stiffness of the artery wall in the state equation, $E_{\text{min}}, E_{\text{max}}, t_m$ – parameters describing the shape of the left ventricular elastance function, R_{lv}, L_{lv} – parameters describing blood flow from the left ventricle to the ascending aorta, L_{la}, R_{la} – parameters describing the blood flow from the left atrium to the left ventricle, V_0 – volume of the left ventricle at zero transmural pressure, $\overline{V_b}$ – the amount of backflow to the left ventricle, S_R, S_C – scaling factors for teminal resistances and compliances, P_{la} – the pressure in the left atrium, and V_{ed} – left ventricular end-diastolic volume.

The analysis was performed for a 45-year old 175 cm tall man with a heart rate (HR) of 75 bpm. We performed the Sobol sensitivity analysis, as described in [21], [22]. All computations were done using the Python library SaLiB v. 1.4.8, [23], [24].

For all analyzed parameters, the initial (nominal) values were taken from the literature and the lower/upper bounds were set at $\pm 50\%$ of the nominal value, except for parameter V_{ed}, for which individual physiological limits were defined (see Table B).

To generate input data, we used a Saltelli sampler [26], which generates $N \cdot (D + 1)$ sets of parameter values, where D is the number of parameters (in our case, $D = 15$), and N is an arbitrary number, preferably a power of 2. The more sets of parameters are generated, the more reliable, although time-consuming, the sensitivity analysis. As a compromise, we took $N = 1024$, which gave us 16384 samples. For each set of parameter values, we simulated the volumetric pulse waves (described in more detail in the next section) in the two arms (radial artery) and the left leg (anterior tibial artery). We decided to use only one leg in the analysis given that the modelled arteries in the two legs are symmetrical. In addition, for each simulation, we also computed stroke volume (SV). To obtain a stable output of the model, we performed 8 seconds of simulation. We present an exemplary output in Fig C.

Table B: **Parameters studied in the sensitivity analysis with adopted limits ($\pm 50\%$ of the nominal values).**

Parameter	Unit	Boundaries	Nominal value	Source of the nominal value
k_1	$\frac{\mathrm{g}}{\mathrm{s}^2 \cdot \mathrm{cm}}$	$\left[1.5 \cdot 10^6, 4.5 \cdot 10^6\right]$	$3 \cdot 10^6$	[25]
k_3	$\frac{\mathrm{g}}{\mathrm{s}^2 \cdot \mathrm{cm}}$	$\left[4.2 \cdot 10^5, 12.6 \cdot 10^5\right]$	$8.4 \cdot 10^5$	computed from [6], for age = 45
E_{max}	$\frac{\mathrm{mmHg}}{\mathrm{ml}}$	$[1.3, 3.8]$	2.5	[4]
E_{min}	$\frac{\mathrm{mmHg}}{\mathrm{ml}}$	$[0.025, 0.074]$	0.049	[4]
t_m	s	$[0.23, 0.68]$	0.45	computed from [4], for HR$= 75$
V_{ed}	ml	$[90, 150]^*$	127	[4]
V_b	ml	$[1, 3]$	2	[4]
V_0	ml	$[5, 15]$	10	[4]
R_{lv}	$\frac{\mathrm{mmHg} \cdot \mathrm{s}}{ml}$	$[0.0167, 0.0501]$	0.0334	[4]
L_{lv}	$\frac{\mathrm{mmHg} \cdot \mathrm{s}^2}{\mathrm{ml}}$	$\left[2.08 \cdot 10^{-4}, 6.24 \cdot 10^{-4}\right]$	0.000416	[4]
R_{la}	$\frac{\mathrm{mmHg} \cdot \mathrm{s}}{\mathrm{ml}}$	$\left[4.45 \cdot 10^{-5}, 1.34 \cdot 10^{-4}\right]$	0.000089	[4]
L_{la}	$\frac{\mathrm{mmHg} \cdot \mathrm{s}^2}{\mathrm{ml}}$	$\left[2.5 \cdot 10^{-5}, 7.5 \cdot 10^{-5}\right]$	0.00005	[4]
P_{la}	mmHg	$[2.5, 7.5]$	5	[4]
S_R	-	$[0.5, 1.5]$	1	assumed
S_C	-	$[0.5, 1.5]$	1	assumed

* individual limits were adopted to obtain physiological values

From the 8 seconds of simulated arterial volume waves, we took the data from the last 1 second sampled at 100Hz, excluding simulations with corrupted results. Then, we conducted the Sobol sensitivity analysis for each considered time point, i.e., $t_1, t_2, \ldots, t_{100}$ separately for waveforms from each limb. The results of this analysis were the first-order sensitivity indices S1 (computed for each studied parameter), which quantify the direct contribution of the given parameter to the model output variability at the given time point of the cardiac cycle. We present the results of this analysis in Fig. D A, C, E. In Fig. D B, D, F, we present the ranked (from smallest to largest) maximal values of S1 for a given limb. A simillar sensitivity analysis was also performed for SP and DP (separately for each limb) and SV. The S1 coefficients for SP, DP, and SV are presented in Fig E. As a cutoff point, we took the value of S1 = 0.05, i.e., we considered the parameters with a maximum S1 coefficient below 0.05 as having little influence on the model output. According to the performed sensitivity analysis, the parameters $E_{\text{min}}, P_{la}, S_R, t_m, E_{\text{max}}$, and k_3 have the highest impact on the studied model outputs (i.e. the arterial volume waveform, SP, DP, and SV) and hence these parameters were included in the identifiability analysis.

4 Parameter identification

To determine possible pairwise correlations between the selected model parameters to limit the number of parameters involved in the patient-specific model optimization, we used the method described by Olufsen et. al., [27]. Let $V_{\text{right arm}}, V_{\text{left arm}}, V_{\text{leg}}$ be arterial volume waveforms in the considered peripheral locations simulated as described in the previous section. To check the local sensitivity of the output $y \in (V_{\text{right arm}}, V_{\text{left arm}}, V_{\text{leg}})$ to the model parameters $\theta = (\theta_1, \ldots, \theta_6) = (E_{\text{min}}, P_{la}, S_R, t_m, E_{\text{max}}, k_3)$, we first determined the relative sensitivity matrix, as follows:

$$\tilde{S} = \frac{\partial y}{\partial \theta} \frac{\theta}{y}, \quad y \neq 0, \tag{18}$$

where

$$\frac{\partial y}{\partial \theta} = \begin{bmatrix} \frac{\partial y}{\partial \theta_1(t_1)} & \cdots & \frac{\partial y}{\partial \theta_6(t_1)} \\ \frac{\partial y}{\partial \theta_1(t_2)} & \cdots & \frac{\partial y}{\partial \theta_6(t_2)} \\ \vdots & \vdots & \vdots \\ \frac{\partial y}{\partial \theta_1(t_{100})} & \cdots & \frac{\partial y}{\partial \theta_6(t_{100})} \end{bmatrix}. \tag{19}$$

To compute the sensitivity matrix we used the central difference approximation, with the steps equal to 0.1% of the nominal value of the given parameter. Then we approximated the model Hessian:

$$H = \tilde{S}^T \tilde{S}. \tag{20}$$

Finally, we calculated the correlation matrix c, where

$$c_{ij} = \frac{C_{i,j}}{\sqrt{C_{ii} C_{jj}}}, \tag{21}$$

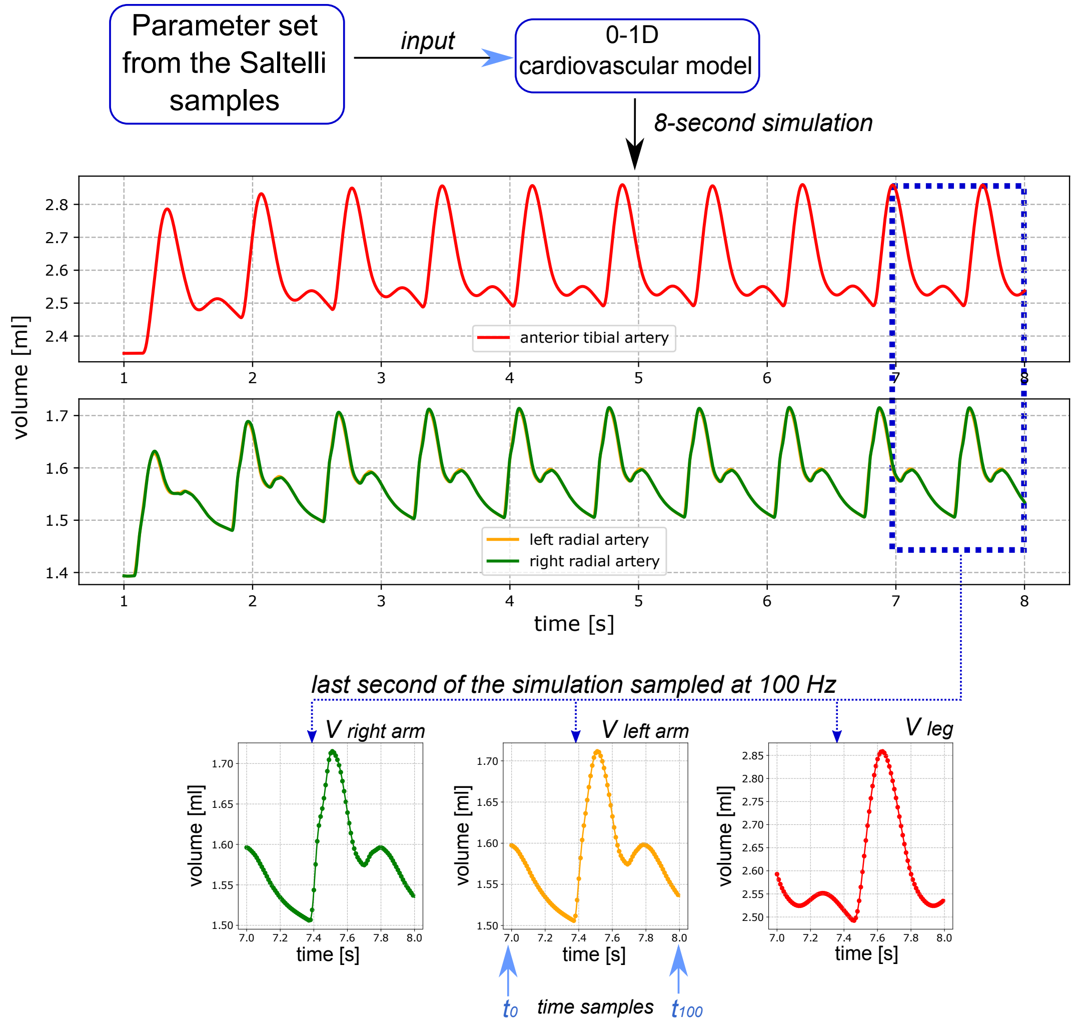

Fig C. **Data processing scheme for the Sobol global sensitivity analysis**. First, we generated 16384 sets of values of 15 parameters (the ranges of parameter values are shown in Table B). Then, for each set of parameter values, using the 0-1D cardiovascular model, we performed 8 seconds simulations of the arterial volume in three analyzed arteries. We used the last second for analysis, sampling the arterial volume waves from the left and right radial artery and anterior tibial artery at 100Hz. We labeled the successive time stamps as $t_0, t_1, \ldots, t_{100}$.

and $C = H^{-1}$ (but only if $\det(H) \neq 0$). A pair of parameters (i, j) is correlated if $|c_{i,j}| > \gamma$ for $\gamma \to 1$. Here, we assumed that two parameters are correlated if $|c_{ij}| > 0.9$. The correlations between parameters can be significantly affected by the values of parameters, for which we compute the corresponding derivatives in the matrix $\tilde{S}$. To understand these correlations better, we computed the coefficients c_{ij} for 150 randomly selected points from the parameter space defined in Table B (assuming uniform distribution of each parameter). Then, for all pairs of parameters, we plotted the coefficients c_{ij} on histograms; see Fig. F. For the vast majority of the generated cases, there was a significant correlation (> 0.9) between the parameters P_{la} and E_{min}. It is noteworthy that in 39% of cases, the correlation was observed only between these two parameters (i.e. $|c_{ij}| > 0.9$ only for the pair E_{min} and P_{la}).

We repeated this procedure in a similar manner for SP, DP (from two arms and one leg) and SV, obtaining a similar relationship between E_{min} and P_{la}. Based on that, we concluded that there is a relationship between E_{min} and P_{la}, and hence we decided to set E_{min} to a fixed value of $0.049\frac{\text{mmHg}}{\text{ml}}$ [4], and use only P_{la} in the optimization process. Hence, we decided that the patient-specific optimization of the model will be performed using the following five parameters: $P_{la}, S_R, t_m, E_{\text{max}}, k_3$. The values of all other parameters studied in the sensitivity analysis were set at their nominal values as shown in Table B.

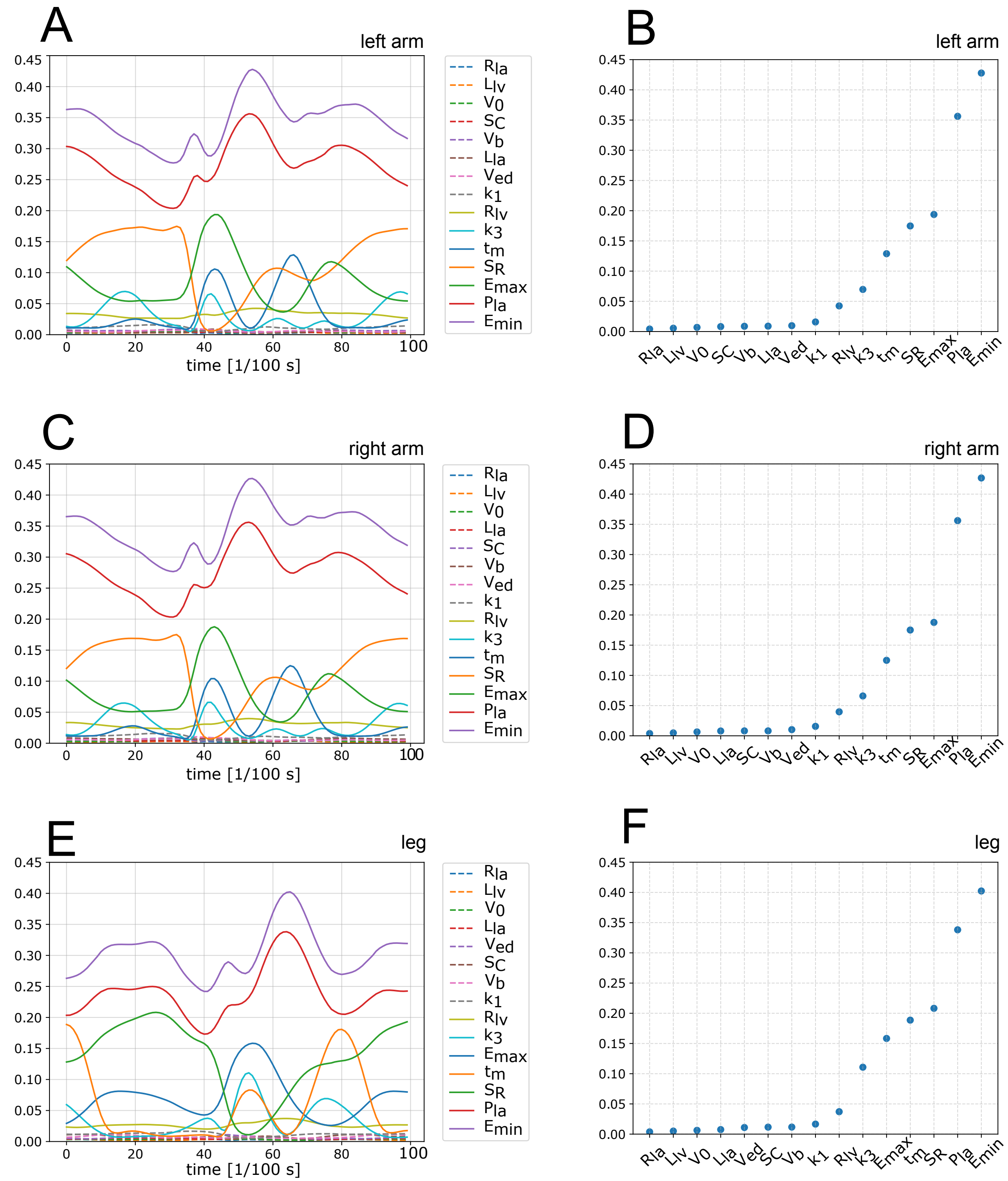

Fig D. **Results of the sensitivity analysis for arterial volume waveforms**. **A, C, E** - time varying values of the S1 index for the last second of the simulation for the left arm, right arm, and leg respectively. **B, D, F** - maximal values of the S1 index computed for the left arm, right arm and leg respectively; since the values of S1 may come from different timestamps, their sum may exceed 1.

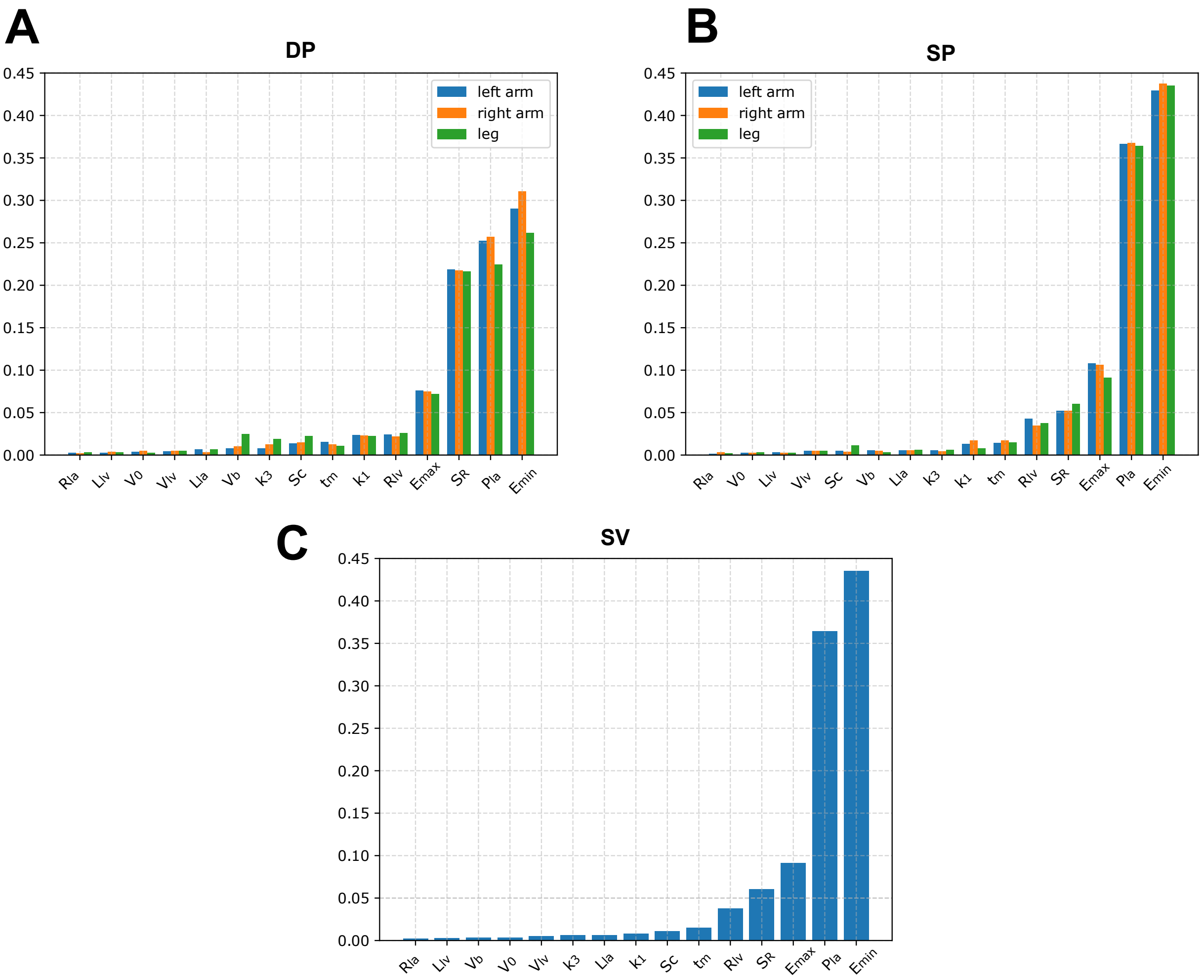

Fig E. **Results of the sensitivity analysis (S1 index) for systolic pressure (A), diastolic pressure (B), and stroke volume (C).**

5 Model optimization

As a result of solving the system of equations described in Section 1, we obtain changes in pressure P, arterial internal cross-sectional area A, and blood flow Q for each point of the 1D arterial domain over time. The model was fitted to pulse waveform data obtained from oscillometric measurements using cuffs on wrists and ankles inflated to a certain level of pressure (see the main text for more details). Since the pulse waves obtained by this method describe changes in blood volume under the cuff, the recorded pulse waveform was fitted by model-simulated changes in the volume of the large artery under the given cuff (e.g. radial artery) V, i.e. the wave computed by integrating the changes in the internal cross-sectional area A over the artery length.

To define the error function to be minimized during the model optimization, first, we normalized in amplitude the recorded and computed pulse waveforms. Then, we approximated the computed waveforms at time points corresponding to the time points of the the recorded waveforms (obtained at 1000 Hz) and compared their differences. Our primary goal was to match the waveform shapes, but to ensure physiological accuracy, we also incorporated into the error function deviations between the diastolic and systolic pressure measured by the AngE device on the left arm (DP_{meas} and SP_{meas}, respectively) and that computed by the model in the left radial artery ($DP_{\text{sim}}, SP_{\text{sim}}$). Additionally, we included a penalty for excessively low (< 40 ml) or high (> 110 ml) stroke volumes, SV_{sim}. Hence, the objective

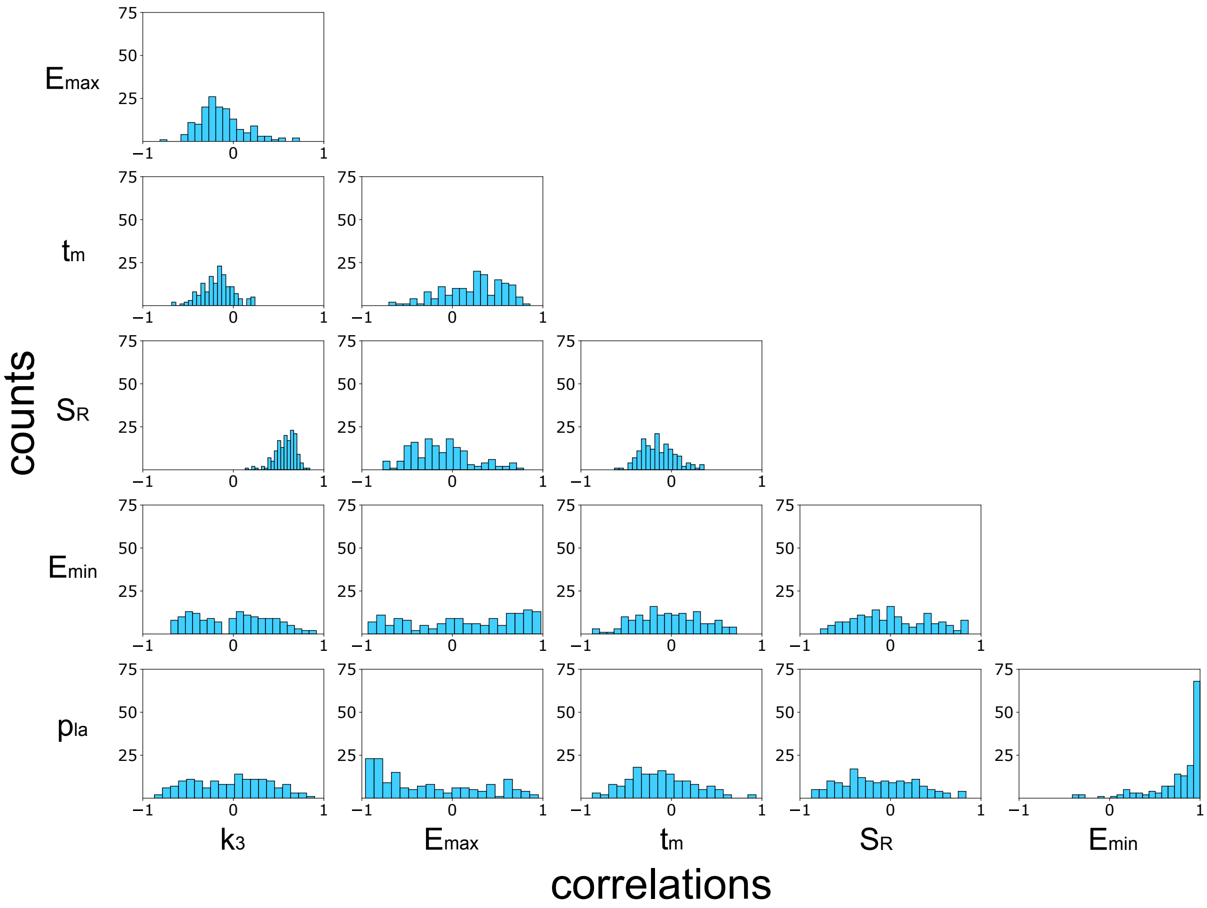

Fig F. **Histograms of correlation coefficients (c_{ij}) for each pair of the studied parameters, generated for 150 randomly selected points from the parameter space**. We set the number of bins in the histograms to 20.

function was as follows

$$\begin{aligned} \text{err} = & \sum_{i \in (\text{left/right arm, left/right leg})} \sum_{j=1}^{n} \|V_{\text{norm, sim; i; j}} - V_{\text{norm, meas; i; j}}\|^2 \\ & + \|DP_{\text{sim}} - DP_{\text{meas}}\|^2/20 + \|SP_{\text{sim}} - SP_{\text{meas}}\|^2/20 + \left((SV_{\text{sim}} - 70)/40\right)^6 , \end{aligned} \quad (22)$$

where *norm*, *sim* and *meas* denotes normalized, simulated and measured values respectively, $\|\cdot\|$ is a ℓ_2-norm, and $n = 1000$ is the number of time points from simulated and measured arterial volume waveforms V. The penalty terms are scaled by fixed values (20 or 40; for all performed fittings) in order to obtain comparable orders of magnitude for first sum and the other terms. The last term is defined in such a way that for SV values between 40 ml and 110 ml it is close to 0, whereas for values smaller or larger the penalty increases exponentially. Indeed, the values of the individual terms may depend on how well the first term is fitted (i.e., the poorer the fit of the normalized waveforms, the more significant the first term). Nevertheless, our priority was to first capture the shape of the volume waves, and only then the pressure values.

To minimize the objective function (22), we used the Levenberg-Marquardt algorithm, [28]. The initial point (i.e. the initial values of the five model parameters being optimized) was set as a combination of parameter values corresponding to the minimal value of the error function amongst the randomly chosen 50 combinations from the parameter space defined in Table B. We present the simplified scheme of the optimization procedure in Fig. 2 in the main text.

References

[1] N. Stergiopulos, D. F. Young, and T. R. Rogge, "Computer simulation of arterial flow with applications to arterial and aortic stenoses," *Journal of Biomechanics*, vol. 25, no. 12, pp. 1477–1488, 1992, ISSN: 0021-9290. DOI: 10.1016/0021-9290(92)90060-E.

[2] M. S. Olufsen, C. S. Peskin, W. Y. Kim, E. M. Pedersen, A. Nadim, and J. Larsen, "Numerical simulation and experimental validation of blood flow in arteries with structured-tree outflow conditions," *Annals of Biomedical Engineering*, vol. 28, no. 11, pp. 1281–1299, 2000, ISSN: 1573-9686. DOI: 10.1114/1.1326031.

[3] J. T. Ottesen, M. S. Olufsen, J. K. Larsen, and M. S. Olufsen, "5. modeling flow and pressure in the systemic arteries," in *Applied Mathematical Models in Human Physiology*, ser. Mathematical Modeling and Computation, Society for Industrial and Applied Mathematics, 2004, pp. 91–136, ISBN: 978-0-89871-539-2. DOI: 10.1137/1.9780898718287.ch5.

[4] J. T. Ottesen, M. S. Olufsen, J. K. Larsen, M. Danielsen, and J. T. Ottesen, "6. a cardiovascular model," in *Applied Mathematical Models in Human Physiology*, ser. Mathematical Modeling and Computation, Society for Industrial and Applied Mathematics, 2004, pp. 137–155, ISBN: 978-0-89871-539-2. DOI: 10.1137/1.9780898718287.ch6.

[5] J. Alastruey, K. H. Parker, J. Peiró, S. M. Byrd, and S. J. Sherwin, "Modelling the circle of Willis to assess the effects of anatomical variations and occlusions on cerebral flows," *Journal of Biomechanics*, vol. 40, no. 8, pp. 1794–1805, 2007, ISSN: 0021-9290. DOI: 10.1016/j.jbiomech.2006.07.008.

[6] P. H. Charlton, J. Mariscal Harana, S. Vennin, Y. Li, P. Chowienczyk, and J. Alastruey, "Modeling arterial pulse waves in healthy aging: A database for in silico evaluation of hemodynamics and pulse wave indexes," *American Journal of Physiology-Heart and Circulatory Physiology*, vol. 317, no. 5, H1062–H1085, 2019. DOI: 10.1152/ajpheart.00218.2019.

[7] H. Suga, K. Sagawa, and D. P. Kostiuk, "Controls of ventricular contractility assessed by pressure-volume ratio, Emax1," *Cardiovascular Research*, vol. 10, no. 5, pp. 582–592, 1976, ISSN: 0008-6363. DOI: 10.1093/cvr/10.5.582.

[8] H. Suga, K. Sagawa, and A. A. Shoukas, "Load independence of the instantaneous pressure-volume ratio of the canine left ventricle and effects of epinephrine and heart rate on the ratio," *Circulation Research*, vol. 32, no. 3, pp. 314–322, 1973, ISSN: 0009-7330. DOI: 10.1161/01.res.32.3.314.

[9] N. Westerhof, G. Elzinga, and P. Sipkema, "An artificial arterial system for pumping hearts," *Journal of Applied Physiology*, vol. 31, no. 5, pp. 776–781, 1971, ISSN: 0021-8987. DOI: 10.1152/jappl.1971.31.5.776.

[10] P. J. Blanco, S. M. Watanabe, E. A. Dari, M. A. R. F. Passos, and R. A. Feijóo, "Blood flow distribution in an anatomically detailed arterial network model: Criteria and algorithms," *Biomechanics and Modeling in Mechanobiology*, vol. 13, no. 6, pp. 1303–1330, 2014, ISSN: 1617-7940. DOI: 10.1007/s10237-014-0574-8.

[11] K. Wołos, L. Pstras, M. Debowska, W. Dabrowski, D. Siwicka-Gieroba, and J. Poleszczuk, "Non-invasive assessment of stroke volume and cardiovascular parameters based on peripheral pressure waveform," *PLOS Computational Biology*, vol. 20, no. 4, e1012013, 2024, ISSN: 1553-7358. DOI: 10.1371/journal.pcbi.1012013.

[12] P. Lax and B. Wendroff, "Systems of conservation laws," *Communications on Pure and Applied Mathematics*, vol. 13, no. 2, pp. 217–237, 1960, ISSN: 1097-0312. DOI: 10.1002/cpa.3160130205.

[13] X. Zhang, S. Noda, R. Himeno, and H. Liu, "Gravitational effects on global hemodynamics in different postures: A closed-loop multiscale mathematical analysis," *Acta Mechanica Sinica*, vol. 33, no. 3, pp. 595–618, 2017, ISSN: 1614-3116. DOI: 10.1007/s10409-016-0621-z.

[14] X. Zhang, D. Wu, F. Miao, H. Liu, and Y. Li, "Personalized hemodynamic modeling of the human cardiovascular system: A reduced-order computing model," *IEEE Transactions on Biomedical Engineering*, vol. 67, no. 10, pp. 2754–2764, 2020, ISSN: 1558-2531. DOI: 10.1109/TBME.2020.2970244.

[15] J. Poleszczuk, M. Debowska, W. Dabrowski, A. Wojcik-Zaluska, W. Zaluska, and J. Waniewski, "Patient-specific pulse wave propagation model identifies cardiovascular risk characteristics in hemodialysis patients," *PLOS Computational Biology*, vol. 14, no. 9, e1006417, 2018, ISSN: 1553-7358. DOI: 10.1371/journal.pcbi.1006417.

[16] J. Poleszczuk, M. Debowska, W. Dabrowski, A. Wojcik-Zaluska, W. Zaluska, and J. Waniewski, "Subject-specific pulse wave propagation modeling: Towards enhancement of cardiovascular assessment methods," *PLOS ONE*, vol. 13, no. 1, e0190972, 2018, ISSN: 1932-6203. DOI: 10.1371/journal.pone.0190972.

[17] H. Senzaki, C.-H. Chen, and D. A. Kass, "Single-beat estimation of end-systolic pressure-volume relation in humans," *Circulation*, vol. 94, no. 10, pp. 2497–2506, 1996. DOI: 10.1161/01.CIR.94.10.2497.

[18] J. D. Parikh, K. G. Hollingsworth, D. Wallace, A. M. Blamire, and G. A. MacGowan, "Normal age-related changes in left ventricular function: Role of afterload and subendocardial dysfunction," *International Journal of Cardiology*, vol. 223, pp. 306–312, 2016, ISSN: 0167-5273. DOI: 10.1016/j.ijcard.2016.07.252.

[19] D. Du Bois and E. F. Du Bois, "A formula to estimate the approximate surface area if height and weight be known," *Archives of Internal Medicine*, vol. XVII, no. 6, pp. 863–871, 1916, ISSN: 0730-188X. DOI: 10.1001/archinte.1916.00080130010002.

[20] V. Bikia et al., "Noninvasive estimation of aortic hemodynamics and cardiac contractility using machine learning," *Scientific Reports*, vol. 10, no. 1, p. 15 015, 2020, ISSN: 2045-2322. DOI: 10.1038/s41598-020-72147-8.

[21] I. M. Sobol', "Global sensitivity indices for nonlinear mathematical models and their Monte Carlo estimates," *Mathematics and Computers in Simulation*, The Second IMACS Seminar on Monte Carlo Methods, vol. 55, no. 1, pp. 271–280, 2001, ISSN: 0378-4754. DOI: 10.1016/S0378-4754(00)00270-6.

[22] A. Saltelli, P. Annoni, I. Azzini, F. Campolongo, M. Ratto, and S. Tarantola, "Variance based sensitivity analysis of model output. Design and estimator for the total sensitivity index," *Computer Physics Communications*, vol. 181, no. 2, pp. 259–270, 2010, ISSN: 0010-4655. DOI: 10.1016/j.cpc.2009.09.018.

[23] T. Iwanaga, W. Usher, and J. Herman, "Toward SALib 2.0: Advancing the accessibility and interpretability of global sensitivity analyses," *Socio-Environmental Systems Modelling*, vol. 4, p. 18 155, 2022. DOI: 10.18174/sesmo.18155.

[24] J. Herman and W. Usher, "SALib: An open-source python library for sensitivity analysis," *The Journal of Open Source Software*, vol. 2, no. 9, 2017. DOI: 10.21105/joss.00097.

[25] J. P. Mynard and J. J. Smolich, "One-dimensional haemodynamic modeling and wave dynamics in the entire adult circulation," *Annals of Biomedical Engineering*, vol. 43, no. 6, pp. 1443–1460, 2015, ISSN: 1573-9686. DOI: 10.1007/s10439-015-1313-8.

[26] A. Saltelli, "Making best use of model evaluations to compute sensitivity indices," *Computer Physics Communications*, vol. 145, no. 2, pp. 280–297, 2002, ISSN: 0010-4655. DOI: 10.1016/S0010-4655(02)00280-1.

[27] M. S. Olufsen and J. T. Ottesen, "A practical approach to parameter estimation applied to model predicting heart rate regulation," *Journal of Mathematical Biology*, vol. 67, no. 1, pp. 39–68, 2013, ISSN: 1432-1416. DOI: 10.1007/s00285-012-0535-8.

[28] J. J. Moré, "The Levenberg-Marquardt algorithm: Implementation and theory," in *Numerical Analysis*, G. A. Watson, Ed., Berlin, Heidelberg: Springer, 1978, pp. 105–116, ISBN: 978-3-540-35972-2. DOI: 10.1007/BFb0067700.
