# Supplementary material for: Personalized pulse wave propagation modeling to improve vasopressor dosing management in patients with severe traumatic brain injury
Source: PLoS Comput Biol. 2025 Sep 15;21(9):e1013501. doi: 10.1371/journal.pcbi.1013501 (PMC12527194; doi:10.1371/journal.pcbi.1013501)
Supplement: S2 File — (PDF) [file pcbi.1013501.s002.pdf]

S2 File: Supplementary Material

for the article

Personalized Pulse Wave Propagation Modeling to Improve Vasopressor Dosing Management in Patients with Severe Traumatic Brain Injury

Kamil Wolos[1], Leszek Pstras[1], Urszula Bialonczyk[1], Malgorzata Debowska[1], Wojciech Dabrowski[2], Dorota Siwicka-Gieroba[2], Jan Poleszczuk[1]

[1]Laboratory of Mathematical Modeling of Physiological Processes
Nalecz Institute of Biocybernetics and Biomedical Engineering
Polish Academy of Sciences, Warsaw, Poland

[2]Department of Anesthesiology and Intensive Therapy,
Medical University of Lublin, Lublin, Poland

In this Supplementary Figures, we show the fits of the model-simulated arterial volume waveforms (shown in black) to the recorded (averaged) waveforms (shown in light blue). All waveforms were normalized in amplitude. For each patient a few cases are shown corresponding to pulse wave recordings performed on different days. For each case, four fits are shown corresponding to four measurement sites: left arm (LA), right arm (RA), left leg (LL), and right leg (RL).

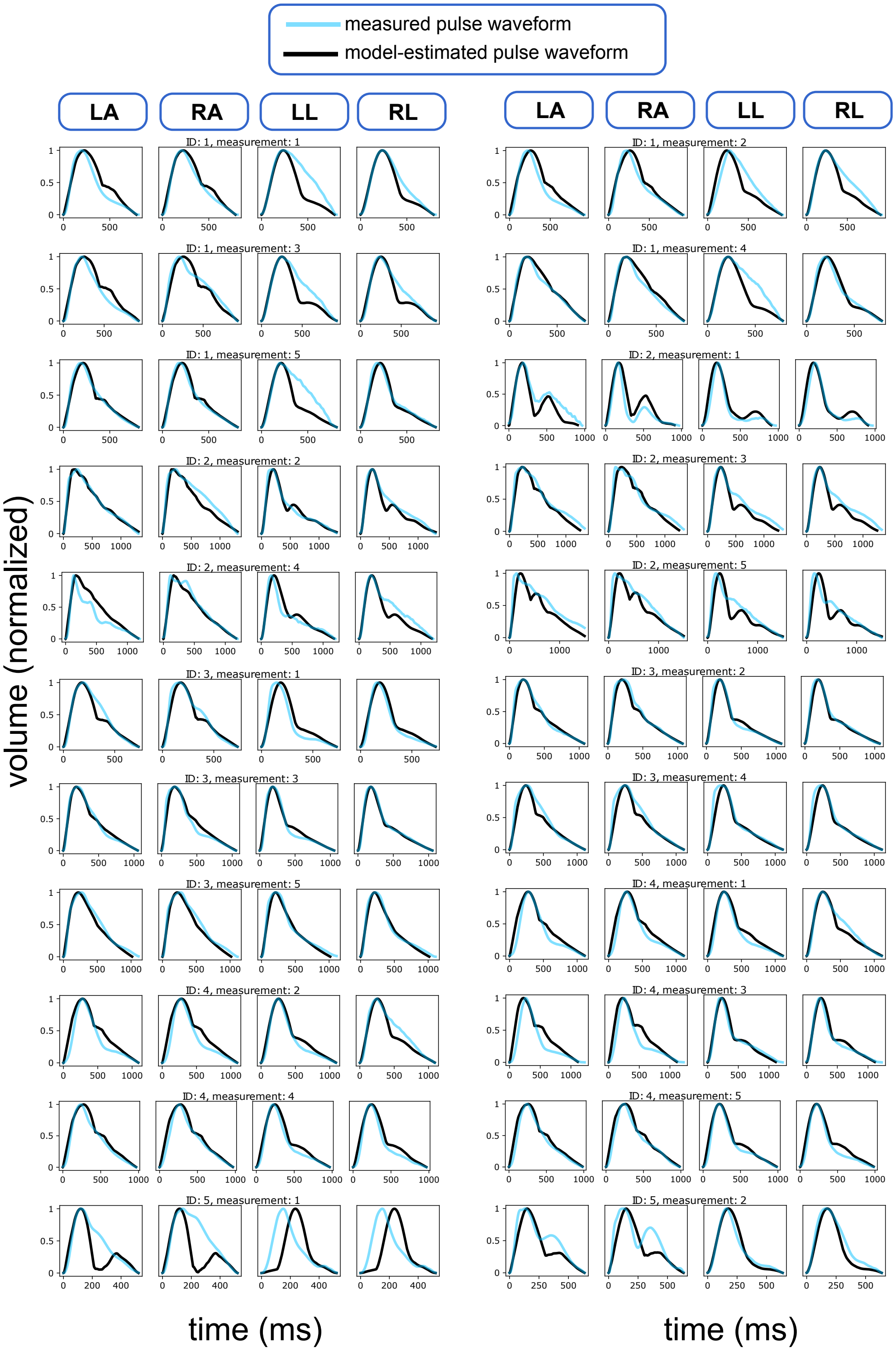

Fig A. Model-simulated vs. recorded arterial volume waveforms

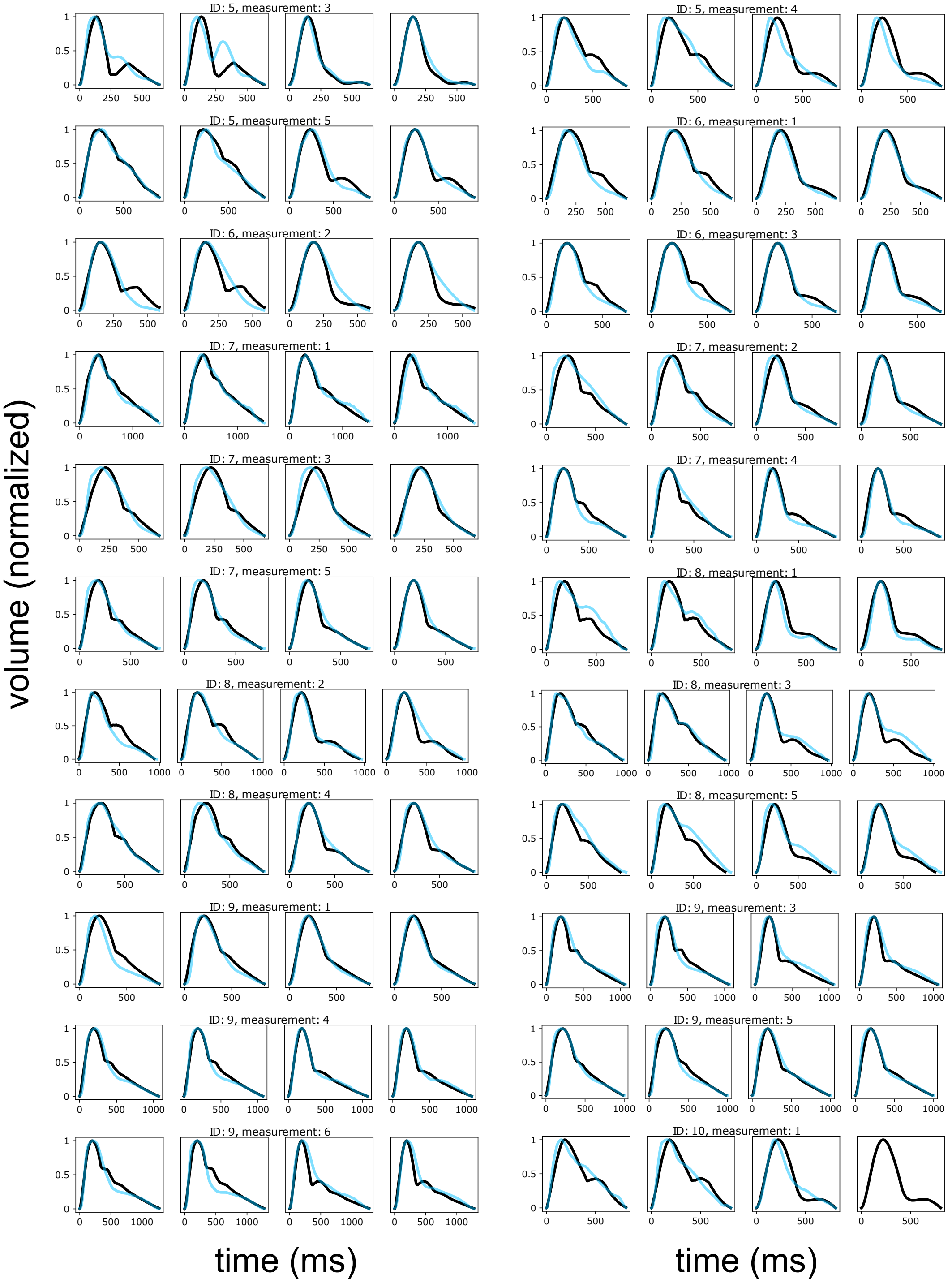

Fig B. Model-simulated vs. recorded arterial volume waveforms

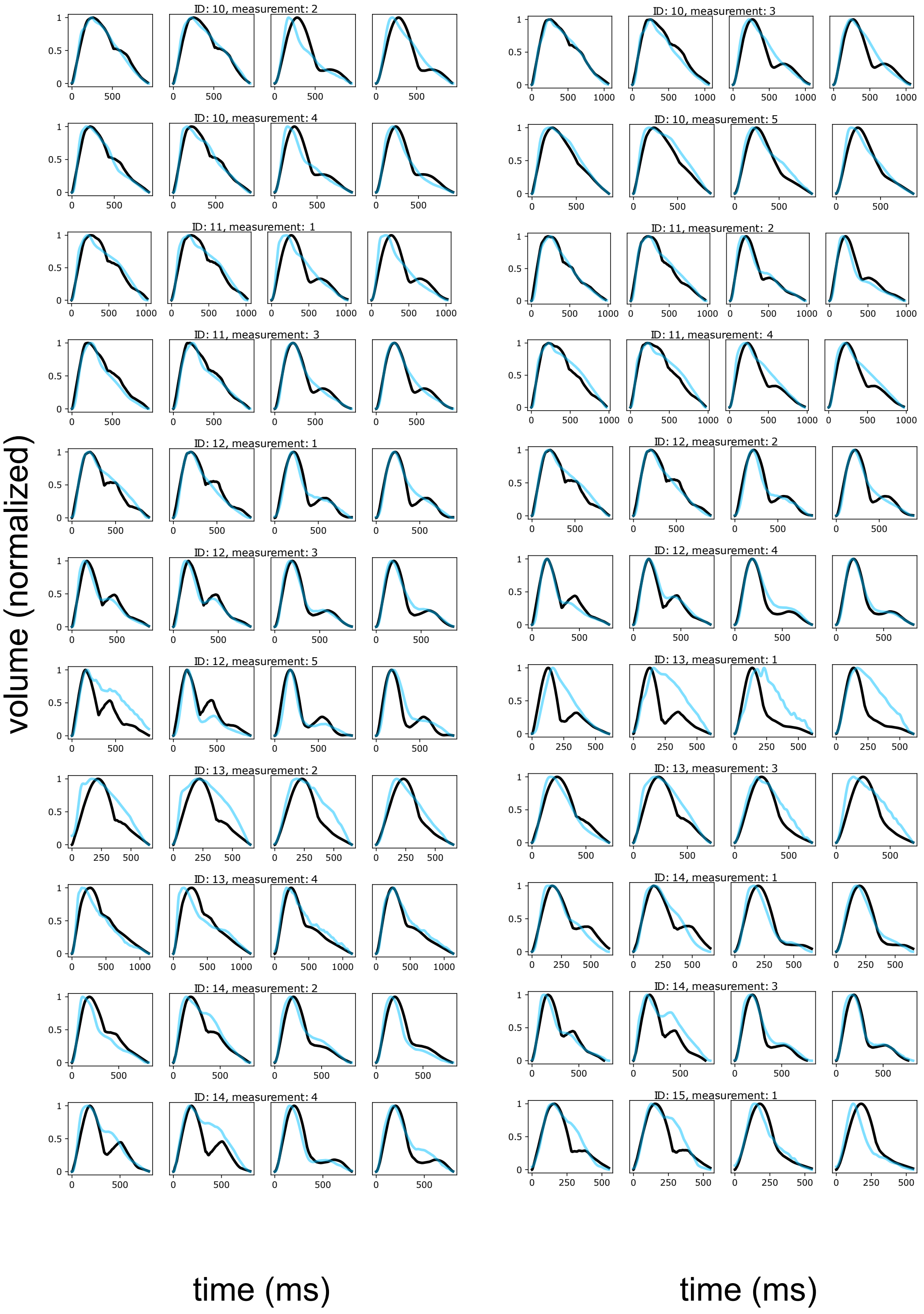

Fig C. Model-simulated vs. recorded arterial volume waveforms

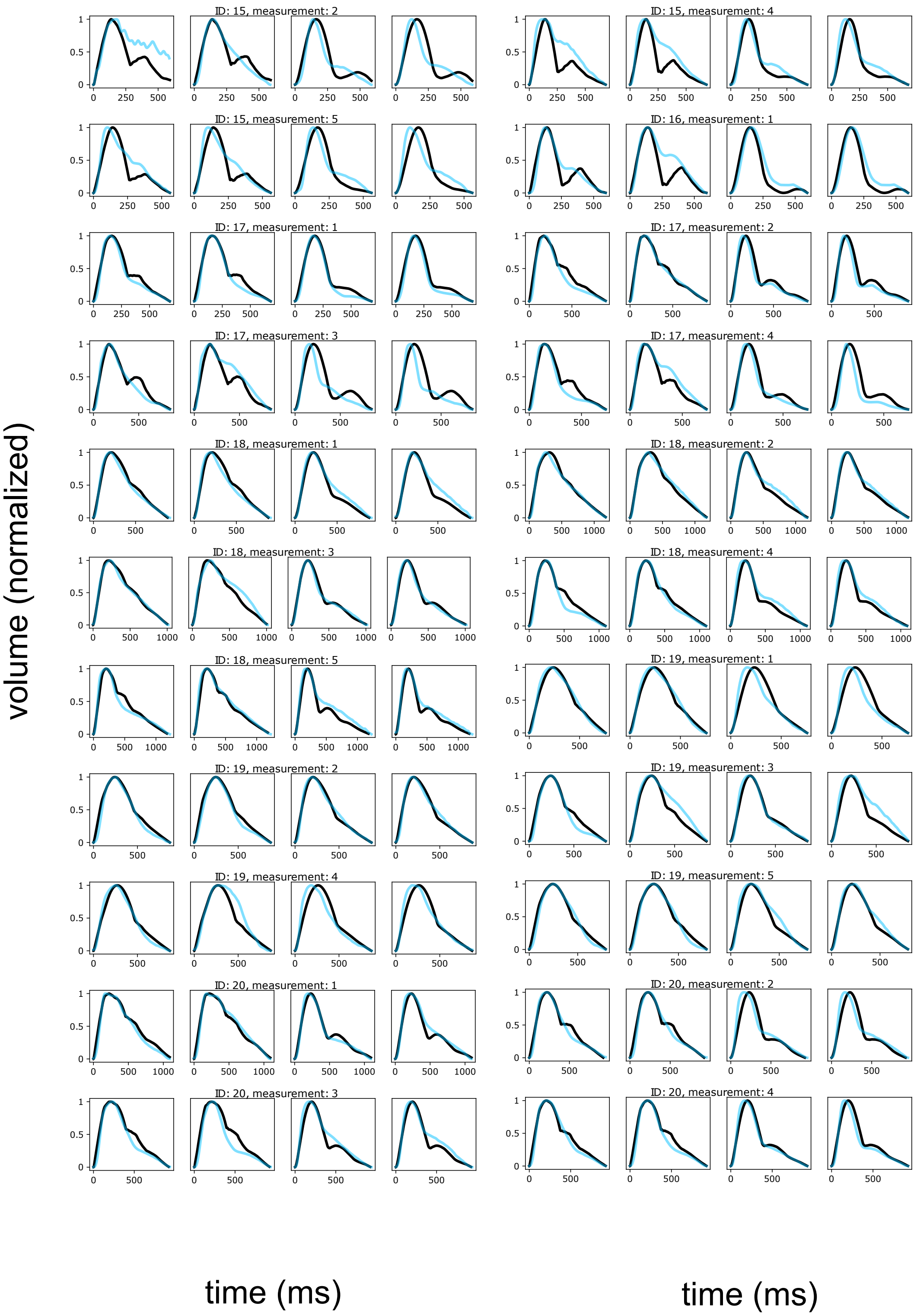

Fig D. Model-simulated vs. recorded arterial volume waveforms
