# Supplementary material for: Personalized pulse wave propagation modeling to improve vasopressor dosing management in patients with severe traumatic brain injury
Source: PLoS Comput Biol. 2025 Sep 15;21(9):e1013501. doi: 10.1371/journal.pcbi.1013501 (PMC12527194; doi:10.1371/journal.pcbi.1013501)
Supplement: S3 File — (PDF) [file pcbi.1013501.s003.pdf]

S3 FILE: SUPPLEMENTARY MATERIAL
FOR THE ARTICLE
PERSONALIZED PULSE WAVE PROPAGATION MODELING TO IMPROVE VASOPRESSOR DOSING MANAGEMENT IN PATIENTS WITH SEVERE TRAUMATIC BRAIN INJURY

Kamil Wolos[1], Leszek Pstras[1], Urszula Bialonczyk[1], Malgorzata Debowska[1], Wojciech Dabrowski[2], Dorota Siwicka-Gieroba[2], Jan Poleszczuk[1]

[1]Laboratory of Mathematical Modeling of Physiological Processes
Nalecz Institute of Biocybernetics and Biomedical Engineering
Polish Academy of Sciences, Warsaw, Poland

[2]Department of Anesthesiology and Intensive Therapy
Medical University of Lublin, Lublin, Poland

This supplement presents changes of measured (on the left wrist cuff; shown in blue) and model estimated (on the radial artery; shown in orange) values of systolic pressure (SP; left column) and diastolic pressure (DP; right column) during the treatment. Additionally, for comparison, the dosage of vasopressors is shown (in light red).

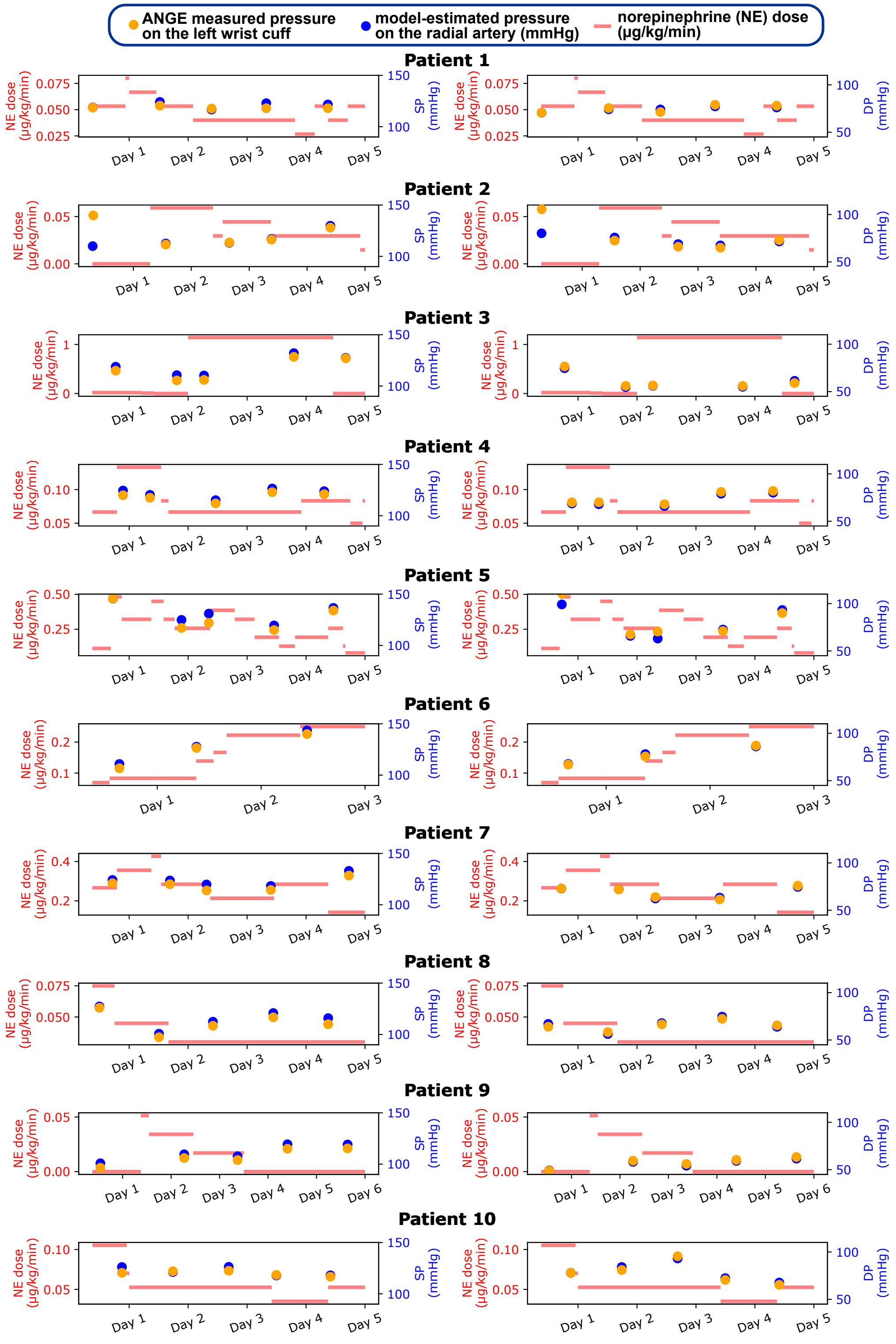

Fig A. Comparison of measured SP and DP (left wrist cuff, blue) and model-estimated values (radial artery, orange) over time during treatment. Vasopressor dosage is indicated in light red for reference (patients 1 – 10).

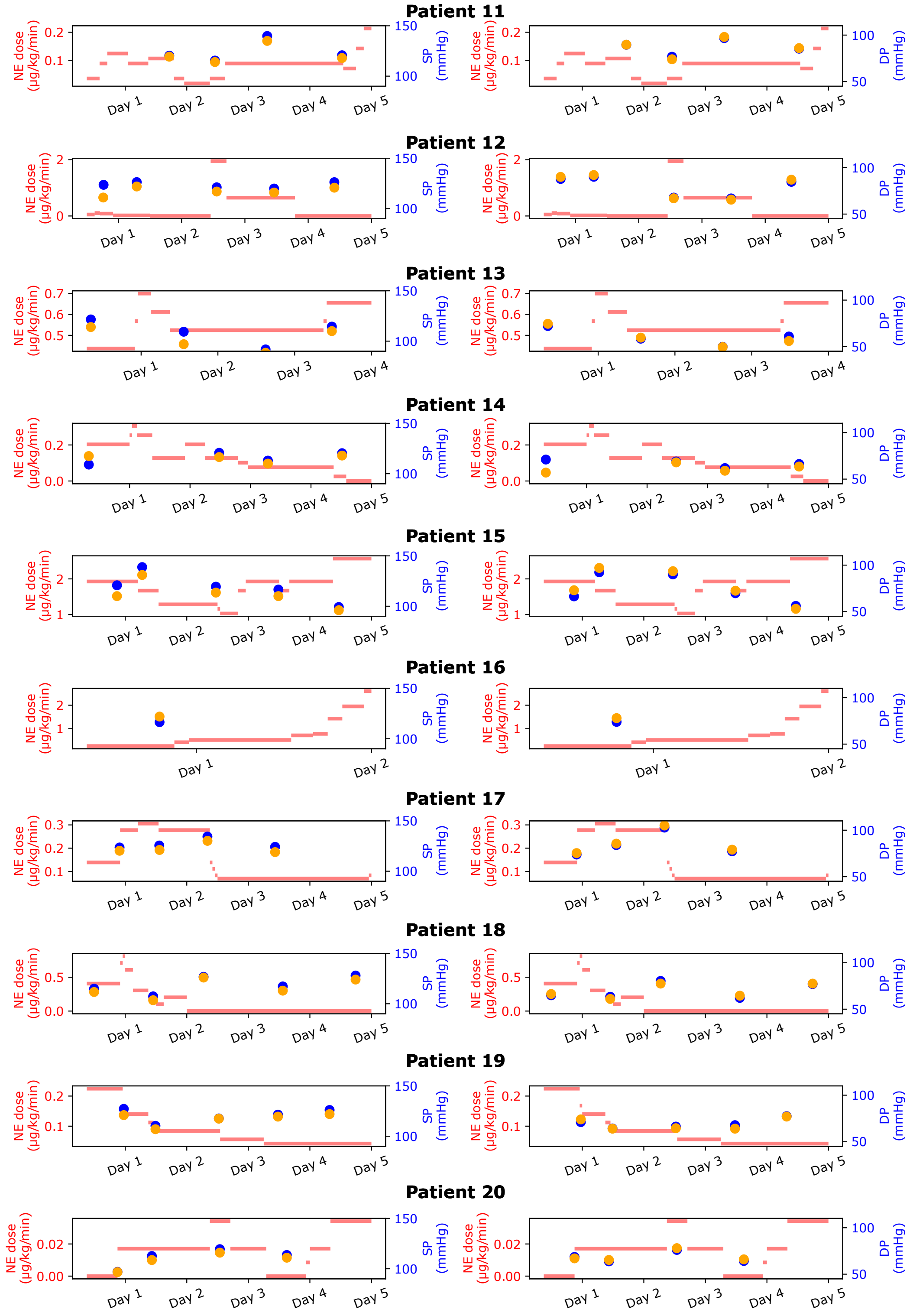

Fig B. Comparison of measured SP and DP (left wrist cuff, blue) and model-estimated values (radial artery, orange) over time during treatment. Vasopressor dosage is indicated in light red for reference (patients 11 – 20).
