# Supplementary material for: Personalized pulse wave propagation modeling to improve vasopressor dosing management in patients with severe traumatic brain injury
Source: PLoS Comput Biol. 2025 Sep 15;21(9):e1013501. doi: 10.1371/journal.pcbi.1013501 (PMC12527194; doi:10.1371/journal.pcbi.1013501)
Supplement: S4 File — (PDF) [file pcbi.1013501.s004.pdf]

S4 File: Supplementary Material
for the article
Personalized Pulse Wave Propagation Modeling to Improve Vasopressor Dosing Management in Patients with Severe Traumatic Brain Injury

Kamil Wolos[1], **Leszek Pstras**[1], **Urszula Bialonczyk**[1], **Malgorzata Debowska**[1], **Wojciech Dabrowski**[2], **Dorota Siwicka-Gieroba**[2], **Jan Poleszczuk**[1]

[1]Laboratory of Mathematical Modeling of Physiological Processes
Nalecz Institute of Biocybernetics and Biomedical Engineering
Polish Academy of Sciences, Warsaw, Poland

[2]Department of Anesthesiology and Intensive Therapy,
Medical University of Lublin, Lublin, Poland

Here, we present a visualization of the vasopressor doses and their adjustments during the observation period, along with the predictions of our statistical model (full model) with regard to the changes in vasopressor dose within the next 24 hours from the time of pulse wave recording. The numbers next to the symbols of predictions indicate the probability assigned to the given prediction.

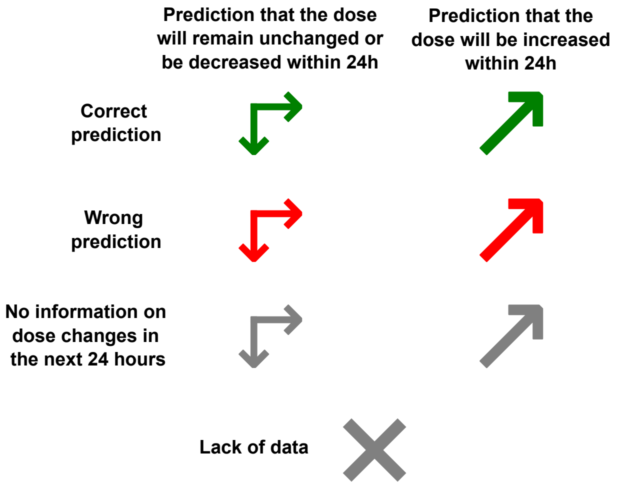

LEGEND

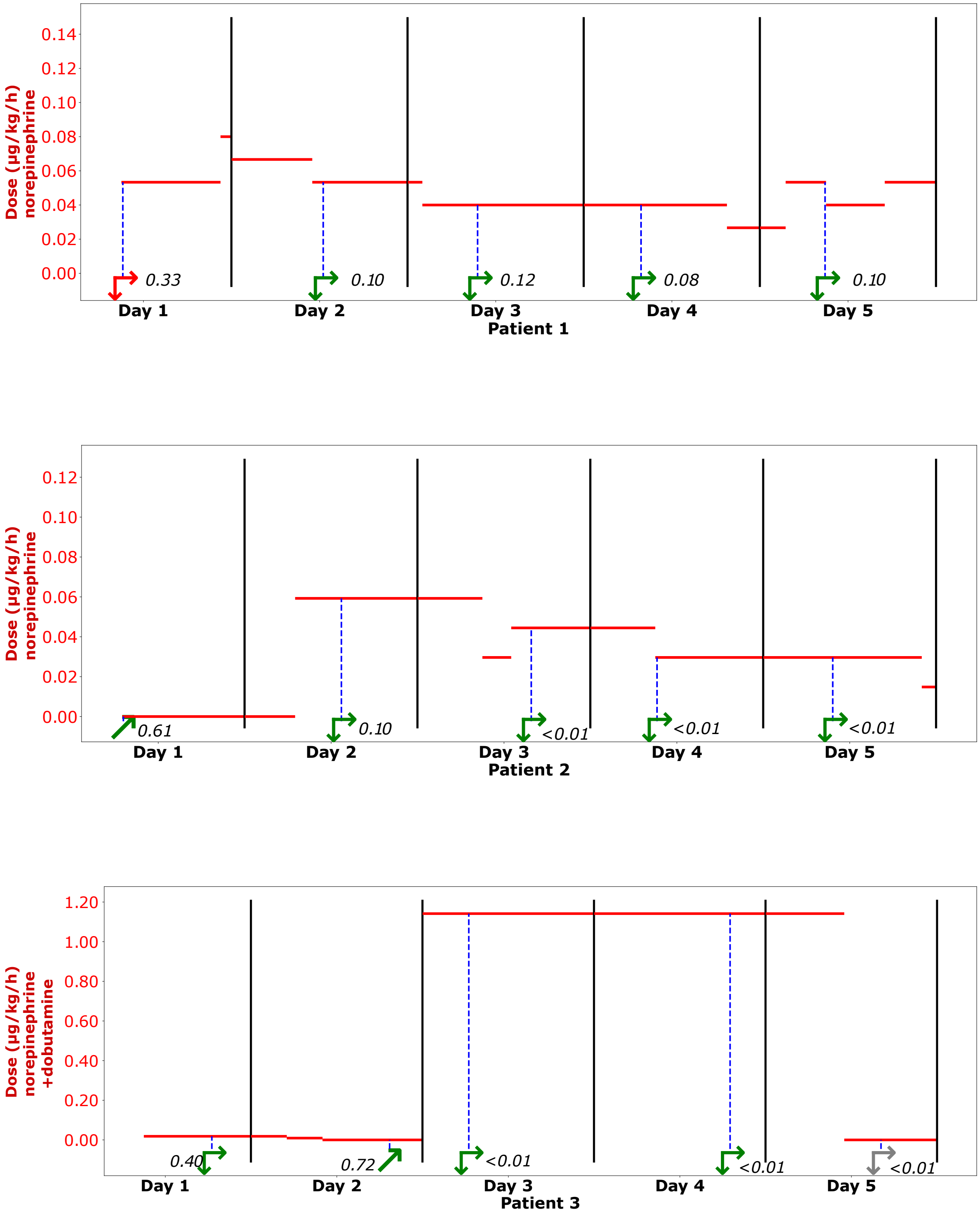

Fig A. Visualization of vasopressor dosage (shown in red) and model predictions for patients 1–3. The full model's predictions for changes in vasopressor dose within the next 24 hours are indicated, with probabilities displayed next to each prediction symbol. Refer to the Legend for further details.

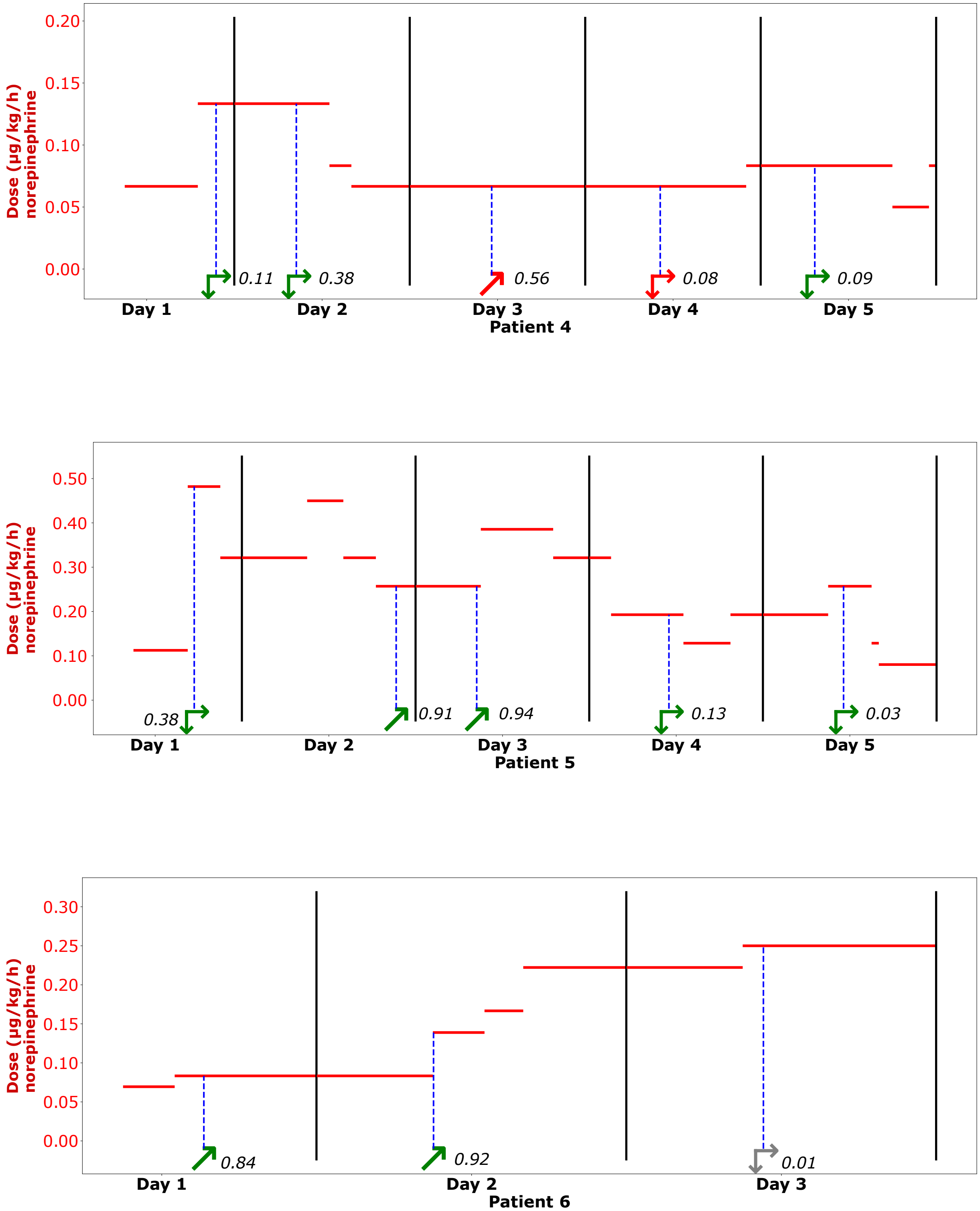

Fig B. Visualization of vasopressor dosage (shown in red) and model predictions for patients 4–6. The full model's predictions for changes in vasopressor dose within the next 24 hours are indicated, with probabilities displayed next to each prediction symbol. Refer to the Legend for further details.

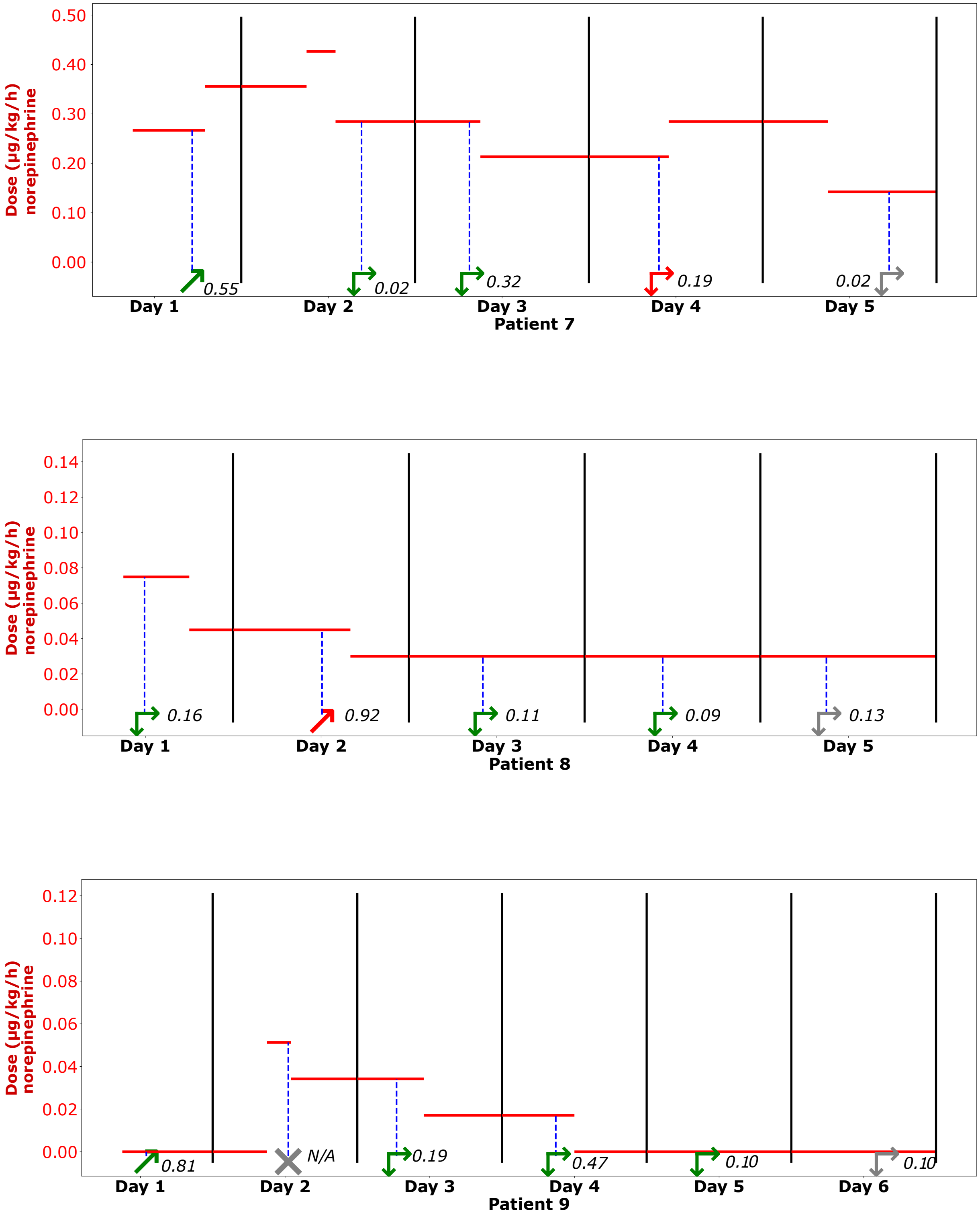

Fig C. Visualization of vasopressor dosage (shown in red) and model predictions for patients 7–9. The full model's predictions for changes in vasopressor dose within the next 24 hours are indicated, with probabilities displayed next to each prediction symbol. Refer to the Legend for further details.

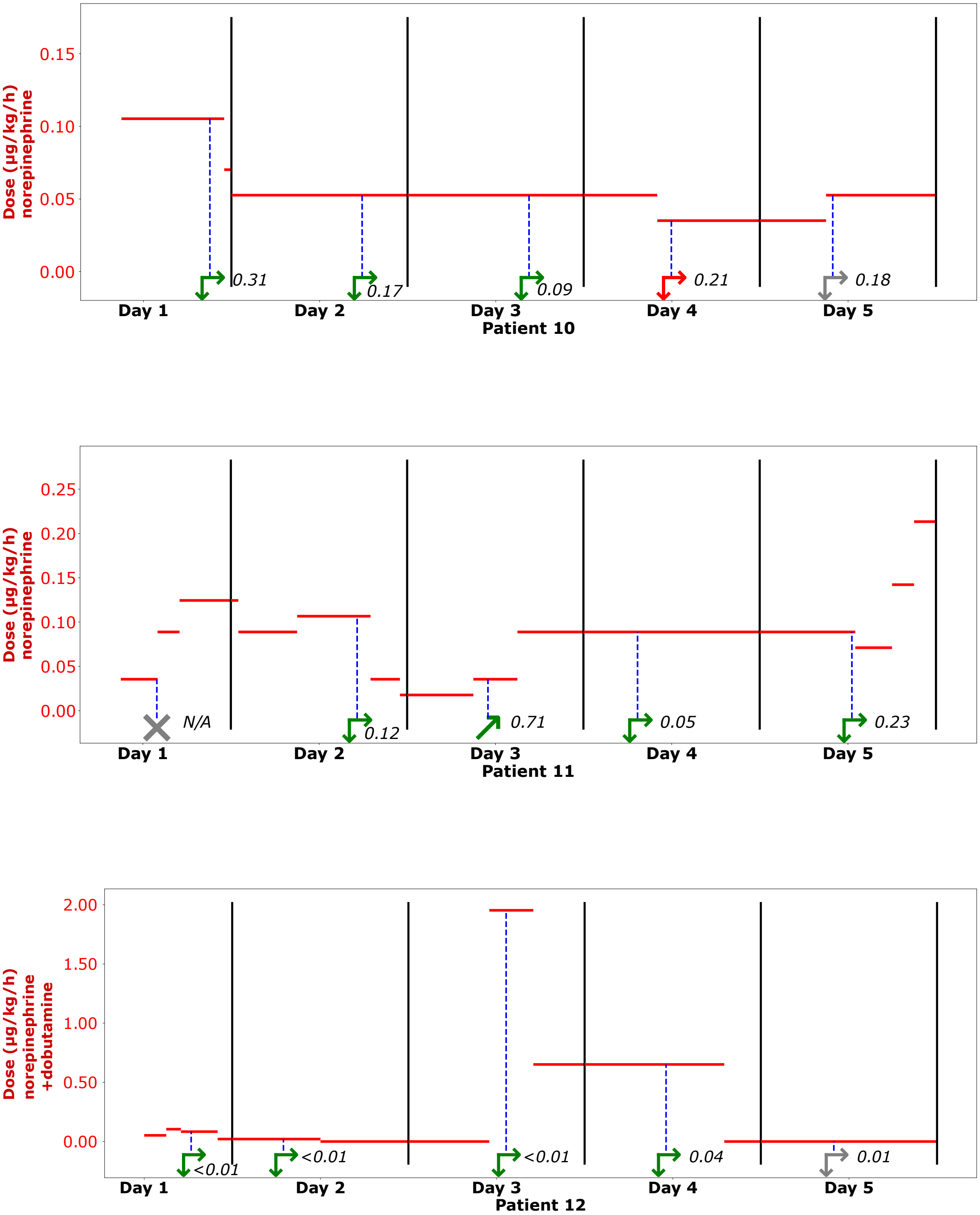

Fig D. Visualization of vasopressor dosage (shown in red) and model predictions for patients 10–12. The full model's predictions for changes in vasopressor dose within the next 24 hours are indicated, with probabilities displayed next to each prediction symbol. Refer to the Legend for further details.

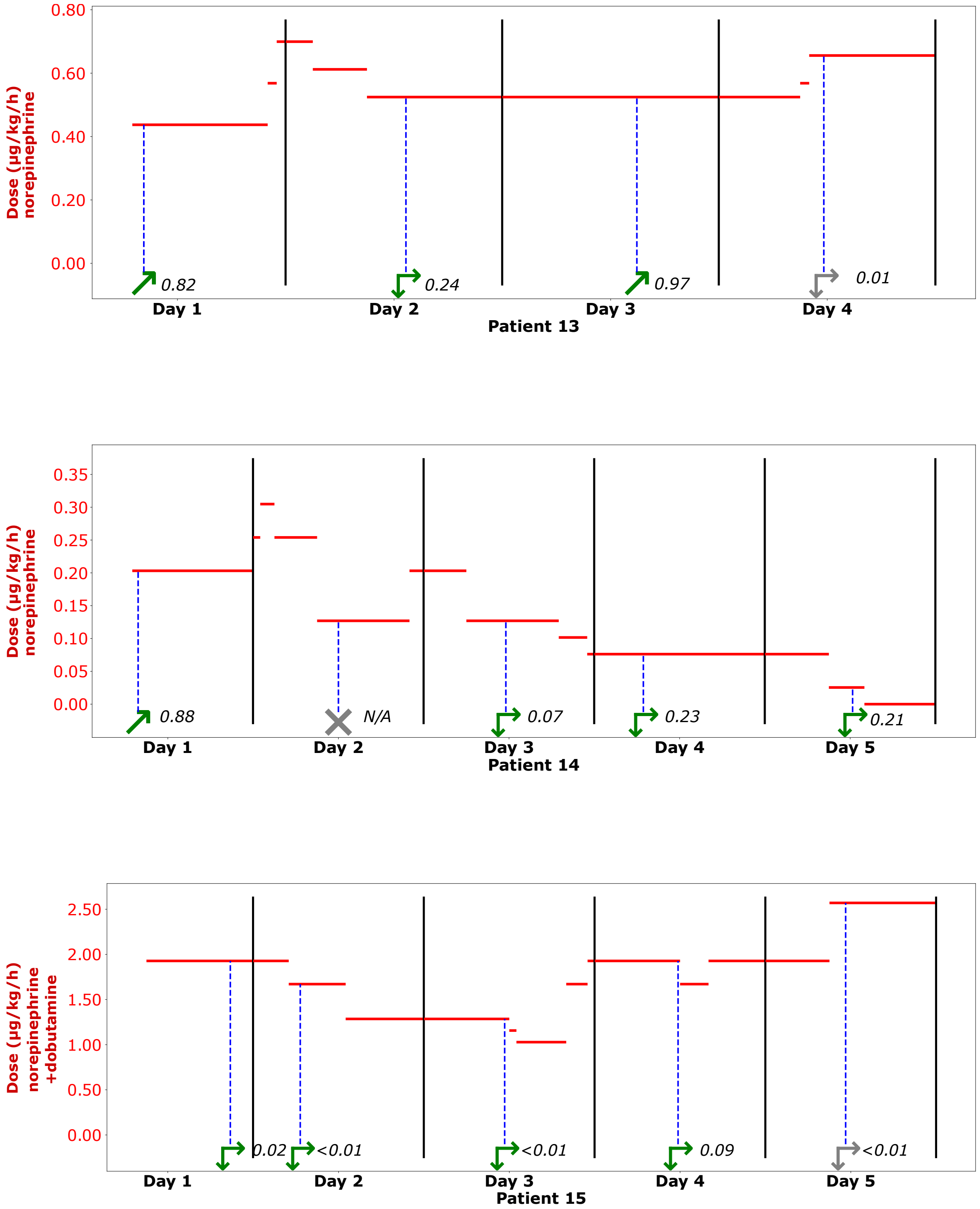

Fig E. Visualization of vasopressor dosage (shown in red) and model predictions for patients 13–15. The full model's predictions for changes in vasopressor dose within the next 24 hours are indicated, with probabilities displayed next to each prediction symbol. Refer to the Legend for further details.

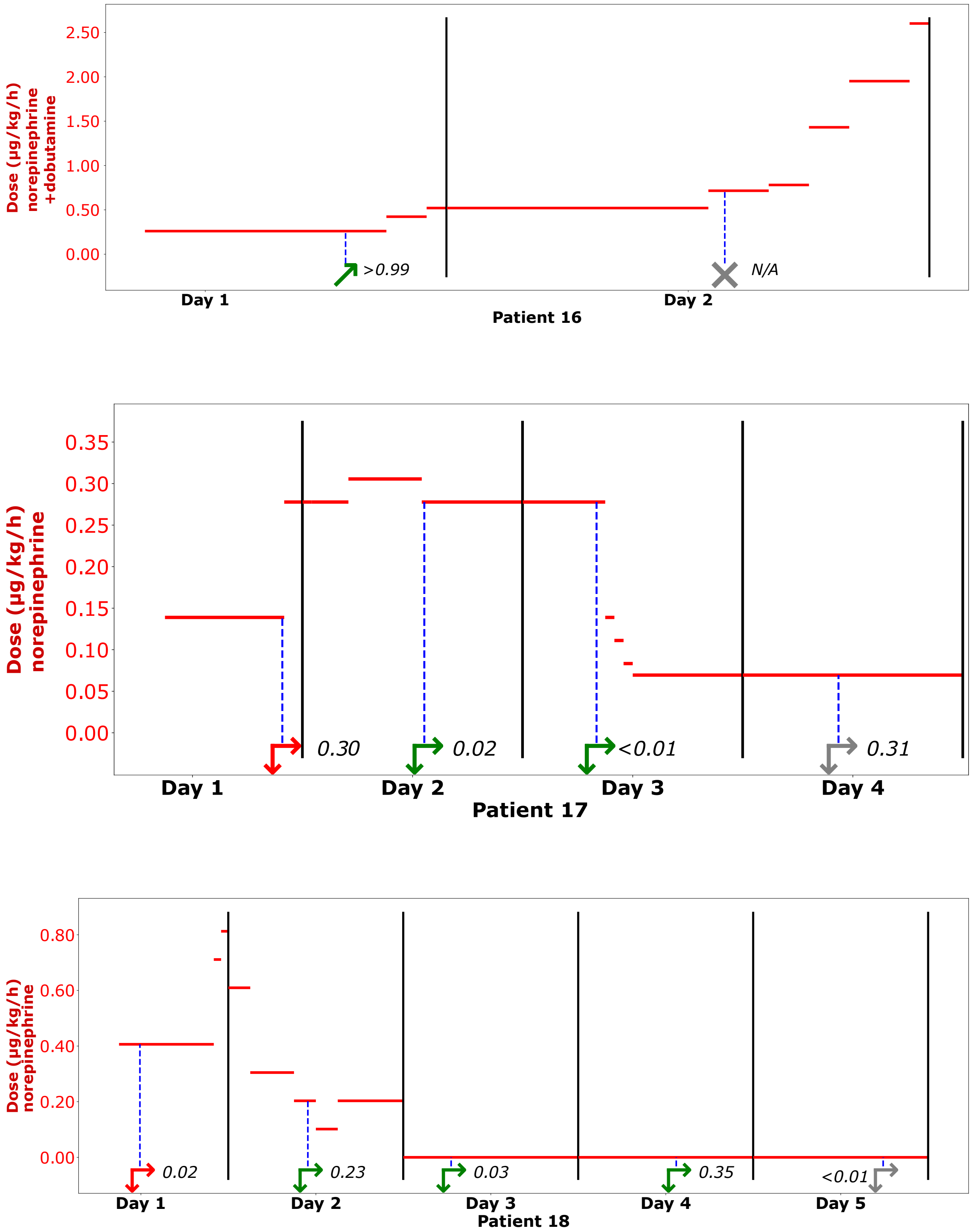

Fig F. Visualization of vasopressor dosage (shown in red) and model predictions for patients 16–18. The full model's predictions for changes in vasopressor dose within the next 24 hours are indicated, with probabilities displayed next to each prediction symbol. Refer to the Legend for further details.

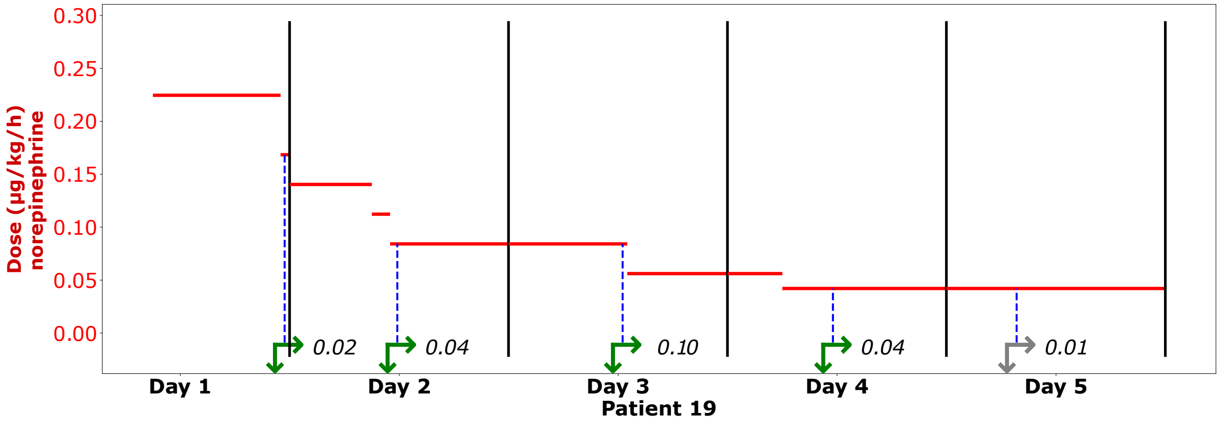

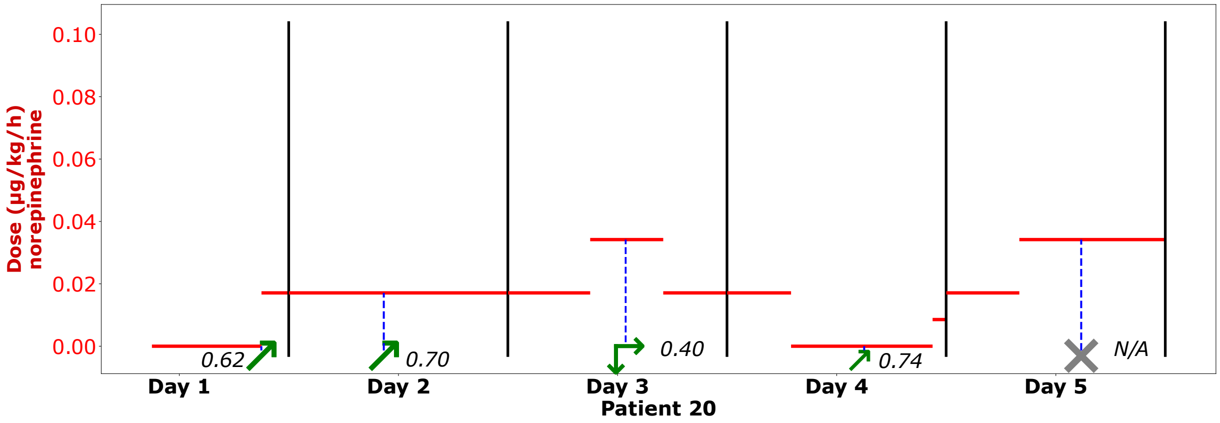

Fig G. Visualization of vasopressor dosage (shown in red) and model predictions for patients 19 and 20. The full model's predictions for changes in vasopressor dose within the next 24 hours are indicated, with probabilities displayed next to each prediction symbol. Refer to the Legend for further details.
